# Synergistic Antibacterial Activity of Fe_3_O_4_@mPEG-Ag Nanoparticles with Molecular Docking Analyses

**DOI:** 10.34133/bmef.0214

**Published:** 2025-12-26

**Authors:** Basit Ali Shah, Hongguo Zhu, Asma Sardar, Yuan Gu, Syed Taj Ud Din, Kashif Naseem, Xinyan Wu, Bin Yuan, Bin Yang

**Affiliations:** ^1^ School of Biomedical Engineering, The Fourth Affiliated Hospital of Guangzhou Medical University, Guangzhou Medical University, Guangzhou 511436, China.; ^2^ School of Materials Science and Engineering, South China University of Technology, Guangzhou 510641, China.; ^3^ Department of Chemistry, Fatima Jinnah Women University, Rawalpindi 46000, Pakistan.; ^4^ Department of Physics, Dongguk University, Seoul 03760, Korea.; ^5^ School of Material and Environmental Engineering, Hunan University of Humanities Science and Technology, Loudi 417000, China.; ^6^ College of Food Science and Nutritional Engineering, China Agricultural University, Beijing 100083, China.

## Abstract

**Objective:** This study aims to develop methoxy poly(ethylene glycol) (mPEG) and silver-modified magnetite nanoparticles termed Fe_3_O_4_@mPEG-Ag NPs as efficient non-antibiotic antibacterial agents to address the growing challenge of drug-resistant bacterial infections. **Impact Statement:** This work demonstrates a synergistic nanomaterial design that achieves high antibacterial efficacy, stability, and biocompatibility, positioning it as a promising alternative to conventional antibiotics in combating antimicrobial resistance. **Introduction:** Infectious diseases caused by drug-tolerant bacteria present a serious global health risk. Fe_3_O_4_@mPEG-Ag NPs were developed as synthetic bactericides that integrate the antibacterial properties of Ag with an excellent stability and dispersibility of mPEG-modified Fe_3_O_4_. **Methods:** Fe_3_O_4_@mPEG-Ag NPs were fabricated via a serial coprecipitation technique. A series of structural and functional characterizations was performed, and antibacterial activity was tested. Additional assessments included minimum inhibitory concentration (MIC) determination, detailed mechanistic evaluation, cytocompatibility assays, and in silico molecular docking studies. **Results:** Fe_3_O_4_@mPEG-Ag NPs demonstrate superior antibacterial activity at a MIC as low as 50 μg·ml^−1^ and achieved an efficacy similar to ciprofloxacin. The improved bactericidal effect is attributed to strong electrostatic interactions, membrane disruption through enhanced reactive oxygen species generation under visible light, and intracellular damage via NP penetration and controlled Ag^+^ leaching. Surface functionalization improves colloidal stability and bioactivity while simultaneously maintaining >80% cell viability. Molecular docking further supports the experimental findings by confirming the inhibition of *Staphylococcus aureus DNA gyrase* and *Escherichia coli β-lactamase* enzymes. **Conclusion:** Fe_3_O_4_@mPEG-Ag NPs demonstrate synergistic antibacterial mechanisms with high biocompatibility, highlighting their potential as effective nanotherapeutics for bacterial control, and represent a promising alternative to conventional antibiotics to combat antimicrobial resistance.

## Introduction

Globally, multidrug-resistant pathogens pose a substantial threat to public health by acquiring resistance to multiple antibiotics, making standard therapies ineffective. Although antibiotics have transformed modern drugs through their life-saving impact, their overuse and stagnant development have driven the rise of antibiotic-resilient pathogens. Some prominent pathogens, including *Pseudomonas aeruginosa*, *Enterococcus faecalis*, *Staphylococcus aureus*, *Enterobacter cloacae*, *Escherichia coli*, *Klebsiella pneumoniae*, and *β-lactamase* producing other Gram-negative bacteria are among the leading causes of persistent and severe infections [1–3]. For instance, merely a single Gram-negative–*K. pneumoniae* bacterium can cause a number of healthcare-associated infections (HAIs), such as liver abscesses, septic arthritis, post-surgical infections, urological infections, and endotracheal tube-related complications, all of which are difficult to treat with conventional antibiotics. It is, therefore, essential to explore innovative, non-antibiotic therapeutic approaches with novel mechanisms to counter the rising risk of antibiotic resistance.

Over the past few decades, inorganic nanomaterials have rapidly emerged as the forefront therapeutic agents in anti-infection research due to their enhanced surface interactions at the nanoscale and their ability to generate excess amount of reactive oxygen species (ROS) [4,5]. Notable examples include metal nanoparticles (NPs), metal oxides and sulfide-based nanomaterials, and various hybrid nanosystems where inorganic nanomaterials are combined with organic or noble metallic materials to enhance their stability and antibacterial functionality. Typically, transition metal oxide (TMO) magnetite NPs (Fe_3_O_4_ NPs) have received considerable attentions for antibacterial therapy owing to their easy laboratory synthesis, high loading/dispersion ability, biocompatibility, environmental safety, and most importantly being inexpensive and sustainability [6,7]. Also, Fe_3_O_4_ nanocomposites exhibit high chemical and structural stability, excellent magnetic properties, visible light activity, and promising electrical performance, making them highly suitable in biomedical fields, ranging from precision drug delivery to bio-separation, photodynamic and photothermal therapies, biosensing, and tissue engineering [8–10]. However, pristine Fe_3_O_4_ NPs face several limitations, including rapid aggregation during synthesis and strong interatomic bonding, which hinder their interaction with bacterial cell walls, while concentration-dependent cytotoxicity to mammalian cells further restricts their widespread biocidal applications. To address these associated challenges, considerable efforts have been devoted, among which surface modification strategies involving polymeric materials are being extensively employed to enhance the antibacterial efficacy and biocompatibility of pristine Fe_3_O_4_ NPs [11]. For instance, coating Fe_3_O_4_ NPs with biopolymers, such as polyaniline, polyvinyl alcohol, methoxy poly(ethylene glycol) (mPEG), chitosan, cellulose, chitin/silk fibroin, and gelatin/alginate, not only enhances colloidal stability and reduces cytotoxicity but also provides a protective barrier that minimizes the metal ion leakage from the magnetite core, thereby improving both safety and long-term functionality [12–16]. Among these, mPEG is particularly noteworthy owing to its strong antibacterial potential, reduced immunogenicity, and effectiveness in preventing bacterial adhesion through the formation of a hydrophilic, stealth-like coating. The high biocompatibility of mPEG greatly ensures minimal adverse effects on healthy tissues, making it an ideal choice for a broad range of biomedical applications, especially in improving the therapeutic efficacy against drug-resistant pathogens. Furthermore, the controlled integration of metallic elements like silver (Ag), palladium (Pd), gold (Au), or ruthenium (Ru) onto the functionalized Fe_3_O_4_ further introduces supplementary antibacterial functionality [17–19], among which Ag NPs are particularly recognized for its broad-spectrum bactericidal performance attributed to the distinct electronic configuration, catalytic behavior, and regulated release of Ag ions (Ag^+^) [20,21]. For example, Nguyen et al. [22] reported the synthesis of Ag–gallium (Ag–Ga) nano-amalgamated particles, in which gallium liquid facilitates galvanic deposition of Ag nanocrystals, enabling controlled Ag^+^ release that enhances antibacterial efficacy while simultaneously reducing cytotoxicity. Another study conducted by the same group revealed that hydroxyapatite (HAp) scaffolds coated with Ag–Ga NPs (HAp–Ag–Ga) exhibited strong antibacterial activity against both Gram-positive and Gram-negative drug-resistant strains due to the multifaceted mechanisms, including reactive species generation, membrane damage, leakage of cytosolic contents, reduced adenosine triphosphate (ATP) level, and disruption of key bacterial processes such as DNA replication, RNA transcription, protein synthesis, and energy metabolism [23]. Similarly, the integration of Ag NPs onto Fe_3_O_4_@mPEG develops a hybrid nanosystem, where the functionalized Fe_3_O_4_ core provides stability and supports controlled Ag^+^ release, contributing to effective bactericidal activation through ROS-mediated oxidative stress and direct membrane disruption. Additionally, by loading Ag NPs, the quantum confinement and surface plasmonic effects contribute to its high photocatalytic performance by enhancing light absorption through shifts in the valence band (VB) and conduction band (CB) positions, ultimately promoting increased separation of photogenerated charge carriers (PCCs) [24]. The enhanced PCC transport in such nanocomposite systems leads to improved photocatalytic efficiency and superior visible light-induced ROS-mediated antibacterial activity. According to the Zhang research group [25], loading Ag NPs onto Fe_3_O_4_@polydopamine enhances the separation of PCCs and significantly improves the photocatalytic antibacterial performance under light irradiation. Similarly, Wang and coworkers [26] developed Ag nanostructure on bioinspired indocyanine green-modified Fe_3_O_4_, demonstrating excellent photo-triggered antimicrobial efficacy toward representative *S. aureus* and *E. coli* strains, resulting from effective PCC transport and ROS generation. Several studies have consistently highlighted the dual-mode antibacterial mechanism of Fe_3_O_4_/Ag NP composites, wherein Fe_3_O_4_ accelerates electron transfer due to its high electrical conductivity (~1.9 × 10^6^ S·m^−1^), while the Ag component functions both as an electron sink to sustain ROS generation and a charge mediator to enhance interfacial charge migration for efficient photo-triggered bacterial eradication [17,24,26,27]. Therefore, the development of polymer-functionalized magnetite NPs with additional bioactive Ag surface modification yields photoresponsive nano-agents with enhanced electrical/thermal conductivity while instantly mitigating the overabundance of Ag^+^ leakage and preventing Fe_3_O_4_ agglomeration, thereby ensuring improved stability and biocompatibility. Previously, our group reported linear polyethyleneimine-coated Ag_2_S_−*x*_/S-C_dots_ hybrid-NPs, which effectively utilized polymeric layers for Ag^+^ stabilization and demonstrated strong antibacterial performance under near-infrared (NIR)-triggered photothermal conditions [28]. However, the dependence on NIR irradiation raised potential safety concerns, such as unintended thermal damage to surrounding healthy tissues, thereby highlighting the need for safer light-responsive alternatives [29]. Although numerous studies have investigated the antimicrobial potential of such nanocomposite materials, their effectiveness in combating multiple drug-resistant pathogens is often constrained by suboptimal synthesis strategies, weak synergetic interactions, and reliance on NIR-based activation, which may pose safety risks and limit overall therapeutic impact. To bridge this gap, our study focuses on investigating the synergistic effects of mPEG coating and Ag loading on the Fe_3_O_4_ core using a facile and cost-effective synthesis technique, aiming to enhance the antibacterial potential of Fe_3_O_4_ NPs. Remarkably, our developed materials achieve enhanced antibacterial efficacy even at low dosages, thereby reducing potential cytotoxicity. This dual-functional modification not only improves particle stability and biocompatibility but also promotes stronger synergistic interaction and efficient radical generation under the safer 430- to 450-nm window of visible light irradiation, offering superior performance in combating a broad spectrum of drug-resistant infectious pathogens compared to previously reported systems.

In this study, we successfully developed mPEG- and Ag-modified Fe_3_O_4_ NPs termed Fe_3_O_4_@mPEG-Ag by employing a serial coprecipitation route to yield highly effective synthetic bactericides. The as-prepared nanomaterials were thoroughly characterized using various spectroscopic and microscopy techniques, and their antibacterial effects on targeted pathogens were then evaluated against several drug-resistant pathogens, including *E. coli* (ATCC 25922), *K. pneumoniae* (ATCC 13883), *S. aureus* (ATCC 25923), and *E. faecalis* (ATCC 19433) under both illuminated/non-illuminated conditions, using a well-known agar well diffusion protocol. The resulting NPs exhibited strong inhibitory activity toward all 4 tested strains, achieving significant inhibition zones at a minimal effective dose of 50 μg·ml^−1^ under 1.6-W visible light source, matching the antibacterial potency of ciprofloxacin (CIP). The killing effect is primarily attributed to synergistic interactions that enable Fe_3_O_4_@mPEG-Ag NPs to anchor onto bacterial cell surfaces, leading to bacterial membrane destabilization through enhanced production of cytotoxic ROS–^•^O_2_^−^, ^•^OH under visible light conditions, combined with their piercing action and controlled Ag^+^ release. The absorption of Ag onto Fe_3_O_4_@mPEG enhanced the material antibacterial efficacy while simultaneously minimizing excessive Ag^+^ leakage, thereby reducing its associated biological toxicity. Finally, we conducted a computational docking analysis (in silico) to evaluate the interaction affinity of Fe_3_O_4_@mPEG-Ag NPs toward *DNA gyrase* (*S. aureus*) and *β-lactamase* (*E. coli*) bacterial enzymes, highlighting their potential as innovative alternative antibacterial agents for combating several drug-resistant pathogens and simultaneously reducing the risks associated with antibiotic abuse. In short, this study supports the advancement of antibacterial nano-agents by exploring the synergistic in vitro antibacterial efficacy and computational docking behavior of Fe_3_O_4_@mPEG-Ag hybrid-NPs, positioning them as a promising non-antibiotic antibacterial for tackling infections caused by drug-resistant pathogens.

## Results and Discussion

### Formation and structural details

**Fig. 1.**
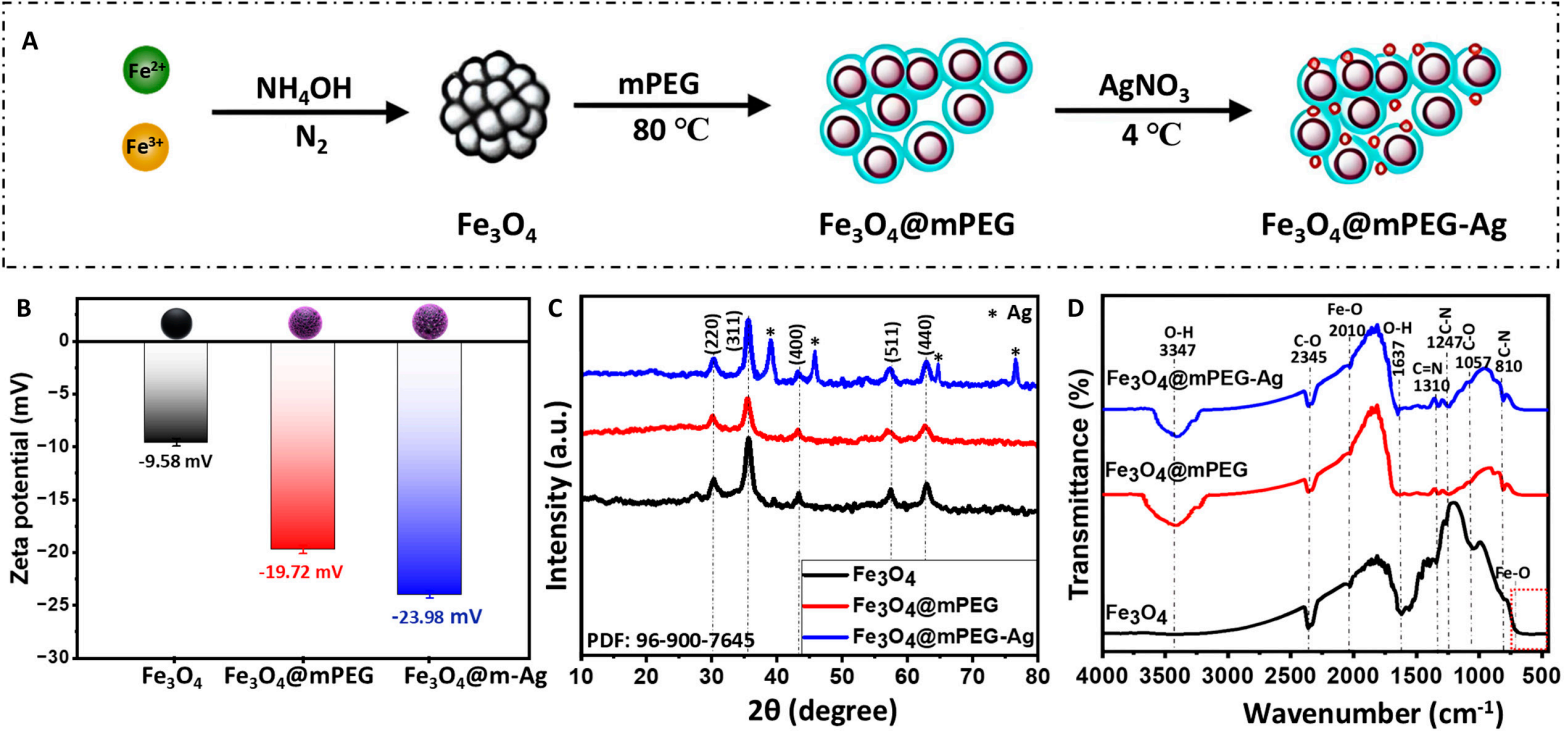
(A) Schematic representation of the formation of Fe_3_O_4_@mPEG-Ag hybrid-NPs. (B) Zeta potential. (C) XRD patterns. (D) FTIR spectra of the prepared materials.

Briefly, Fe_3_O_4_ and Fe_3_O_4_@mPEG counterparts were first prepared using a serial one-step redox reaction synthesis method, followed by Ag loading to develop resulting Fe_3_O_4_@mPEG-Ag hybrid-NPs as outlined in the graphical depiction of Fig. 1A. During the fabrication, hydroxyl and ether functional groups present on the Fe_3_O_4_@mPEG surface actively contributed to the reduction and deposition of Ag^+^, functioning as both reducing and stabilizing agents. This process transforms Ag^+^ into metallic Ag NPs while forming stable coordination linkages. Additionally, the mPEG coating creates a hydrophilic protective layer that prevents Fe_3_O_4_ NP aggregation and ensures uniform Ag NP distribution with enhanced interfacial stability. As depicted in Fig. 1B, the formation of Fe_3_O_4_@mPEG-Ag NPs was confirmed by zeta potential analysis, which showed a progressive decrease in surface charge values across the synthesis steps. The bare Fe_3_O_4_ NPs exhibited a zeta potential of approximately −9.58 mV, indicating moderate colloidal stability, while upon mPEG functionalization and subsequent Ag loading, further reductions in the zeta potential were observed, suggesting effective surface charge neutralization and increased structural complexity. This progressive decline confirms the stepwise surface modification and successful formation of the resultant hybrid-NPs. The XRD characterization of the as-developed Fe_3_O_4_@mPEG-Ag hybrid-NPs was conducted and compared with those of Fe_3_O_4_@mPEG and Fe_3_O_4_ counterparts (Fig. 1C). The XRD pattern of the pristine Fe_3_O_4_ displays the characteristic peaks positioned at 2θ values of 30.89°, 35.34°, 44.75°, 58.13°, and 64.18° corresponding to the (220), (311), (400), (511), and (440) planes of the fcc (face-centered cubic) crystal structure of Fe_3_O_4_ (JCPDS #96-900-7645) [30]. Similar diffraction peaks were observed in both Fe_3_O_4_@mPEG and Fe_3_O_4_@mPEG-Ag NP samples showing a marked decrease in peak intensities relative to pristine Fe_3_O_4_, while additional peaks at 2θ of 38.14° [Ag (111)], 44.23° [Ag (200)], 64.71° [Ag (220)], and 77.93° [Ag (302)] confirm the incorporation of metallic Ag in the Fe_3_O_4_@mPEG-Ag hybrid-NP sample [17,31]. The reduced peak intensities in both Fe_3_O_4_@mPEG and Fe_3_O_4_@mPEG-Ag NP samples can be attributed to the amorphous nature of the mPEG, characterized by the absence of long-range atomic ordering, thereby diminishing the diffraction intensity compared to crystalline materials. This phenomenon has been consistently observed when polymeric coatings are introduced onto crystalline materials, resulting in reduced XRD peak intensities due to the noncrystalline nature of the coating shell [12,32,33]. To further confirm the formation of Fe_3_O_4_@mPEG-Ag hybrid-NPs, FTIR analysis was piloted to identify distinct surface functional groups present in the corresponding samples. The spectra shown in Fig. 1D display distant vibrational patterns corresponding to Fe_3_O_4_, Fe_3_O_4_@mPEG, and Fe_3_O_4_@mPEG-Ag bands, confirming the successful integration of the functional components. The FTIR analysis of the pristine Fe_3_O_4_ NPs displays prominent peaks, including broad and intense Fe–O vibrational modes observed in the 550 to 690 cm^−1^ and 2,010 cm^−1^ regions, indicating the metal–organic framework. Two peaks positioned at 1,057 and 2,345 cm^−1^ are linked to C–O stretching within benzene ring compounds, whereas the bands at 1,247 and 1,310 cm^−1^ are associated with C–N and C=N stretching in aromatic and aliphatic amines [34]. Additionally, O–H stretching vibrations of adsorbed water are detected at 1,637 cm^−1^ and beyond 3,000 cm^−1^, ensuring surface hydration [17,31,35]. The characteristic bands of the Fe_3_O_4_ are retained in both Fe_3_O_4_@mPEG and Fe_3_O_4_@mPEG-Ag NP samples, indicating the structural integrity of the magnetite core. Notably, a broad O–H stretching vibration of adsorbed water band at around 3,347 cm^−1^ exhibits a slight shift toward lower wavenumbers in the Fe_3_O_4_@mPEG-Ag NP sample compared to Fe_3_O_4_@mPEG, indicating stronger hydrogen bond interactions due to the presence of Ag NPs [31]. Furthermore, the O–H bending vibration mode at 1,637 cm^−1^ becomes more intense in the resultant Fe_3_O_4_@mPEG-Ag hybrid-NP sample and demonstrates a shift slightly toward higher wavenumbers in both Fe_3_O_4_@mPEG and Fe_3_O_4_@mPEG-Ag NP samples compared to a pristine Fe_3_O_4_ sample. This observation reflects enhanced water molecule adsorption and strengthened interfacial interactions resulting from the mPEG coating and subsequent Ag-loading modifications. Overall, the FTIR findings confirm the successful formation of the Fe_3_O_4_@mPEG-Ag hybrid-NPs with evident structural and chemical characteristics aligning well with the XRD results.

### Morphological and elemental mapping analyses

**Fig. 2.**
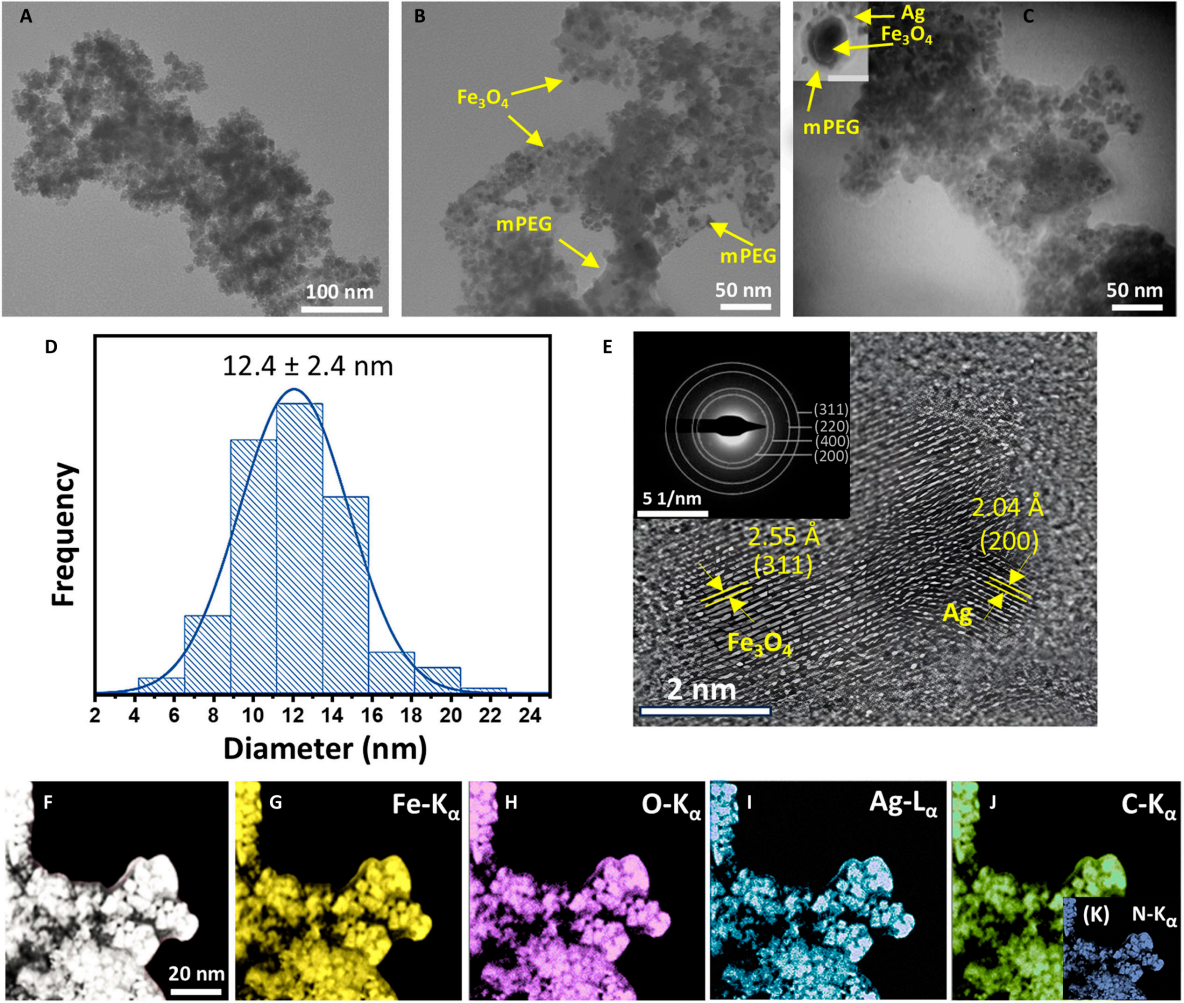
TEM micrographs of the (A) pristine Fe_3_O_4_, (B) Fe_3_O_4_@mPEG, and (C) Fe_3_O_4_@mPEG-Ag hybrid-NPs with an inset image at 10 nm. (D) Histogram with size distribution. (E) HRTEM image with Saeed patterns (inset) of the Fe_3_O_4_@mPEG-Ag NPs. (F) HAADF-STEM image of the Fe_3_O_4_@mPEG-Ag hybrid-NPs, with its (G to K) corresponding elemental mapping images.

The structural morphologies and elemental distribution of the well-prepared NPs were investigated through TEM/high-resolution TEM (HRTEM), HAADF-STEM, and EDS elemental mapping analysis, as illustrated in Fig. 2A to J. The TEM micrograph of the pristine Fe_3_O_4_ sample (Fig. 2A) displays nanosized particles with a roughly spherical morphology, appearing indistinct and clouded due to the particle aggregation effect. A noticeable degree of particle clustering can be attributed to the natural magnetic dipole–dipole interactions characteristic of the magnetite–Fe_3_O_4_ NPs, which promote aggregation under normal conditions. This phenomenon is commonly observed in magnetic nanomaterials due to their high surface energy and mutual magnetic attraction attributes [25–27,36]. Furthermore, the relatively uniform particle size distribution suggests a controlled synthesis method; however, the intrinsic tendency of the Fe_3_O_4_ NP agglomeration crucially needs the surface modification of the NPs to enhance their colloidal stability. To tackle this challenge, the morphology depicted in Fig. 2B reveals a substantial improvement in particle dispersion, with the NPs appearing well-distributed and encapsulated within the mPEG-coated layer. This coating effectively mitigates magnetic dipole interactions by introducing a hydrophilic barrier, which not only reduces particle aggregation and enhances colloidal stability but also provides active surface sites for subsequent nanomaterial loading or functionalization, thereby developing advanced hybrid nanostructures with improved properties. The TEM image in Fig. 2C provides a clearer view of Ag-loaded Fe_3_O_4_@mPEG hybrid-NPs, revealing well-dispersed and irregular spherical-shaped NPs with an estimated particle size range of 10 to 20 nm. The uniform distribution of these hybrid-NPs further confirmed that the mPEG coating effectively prevents the NP aggregation and facilitates the smooth incorporation of the Ag NPs. A slight degree of aggregation or clustering observed in Fig. 2C can be partially attributed to the TEM sample preparation, where solvent evaporation during grid drying induces capillary forces that bring NPs into closer proximity. This minor clustering is a common fact in ultrasmall nanomaterials and may not reflect colloidal instability. Importantly, it promotes intimate interfacial contact between Fe_3_O_4_ and Ag domains, thereby enhancing charge transfer and redox coupling between both components, which is crucial for photocatalytic potential. Notably, the presence of mPEG and Ag NPs on the Fe_3_O_4_ surface is indicated by their distinct color contrast and particle sizes, as shown in the inset of Fig. 2C, highlighting the successful integration of all 3 components in the resultant hybrid-NP system. The hybrid-NPs exhibited an average particle size of approximately 12.4 ± 2.4 nm (Fig. 2D), which closely correlates with the TEM observed size range of 10 to 20 nm, confirming a narrow size distribution and good colloidal stability. DLS analysis further ensured this uniformity, indicating high colloidal stability and supporting that the minor clustering observed in the TEM images primarily arises from drying effects of the TEM grid rather than intrinsic aggregation in solution. Furthermore, the HRTEM image (Fig. 2E) confirms the crystalline structure of the Fe_3_O_4_ and Ag components in the hybrid-NPs. The lattice fringes with a spacing of *d* = 2.55 ± 0.13 Å correspond to the (311) plane of Fe_3_O_4_, while the spacing of *d* = 2.04 ± 0.08 Å aligns with the (200) plane of Ag. These findings are further corroborated by the SAED pattern (inset of Fig. 2E), which displays concentric diffraction rings matching the (311), (220), and (400) planes of Fe_3_O_4_, and the (200) plane of Ag, which are consistent with the findings from the XRD analysis. Moreover, HAADF-STEM analysis in Fig. 2F reveals detailed structural insight into the Fe_3_O_4_@mPEG-Ag hybrid-NPs, highlighting their fine structural features and ensuring a relatively uniform morphology. The bright contrast further distinguishes the distribution of all elements in the hybrid structure, indicating their successful incorporation. The corresponding EDX elemental mapping micrographs (Fig. 2G to K) demonstrate a consistent distribution of the Fe, O, Ag, C, and N elements in the hybrid-NPs. In addition, the EDS information in Fig. S1 provides additional confirmation of the presence of the anticipated elemental signals in the resulting sample spectrum, accompanied by an inset table outlining the atomic and weight percentages of each elemental composition. These findings provide convincing evidence confirming the morphological structure integrity and elemental distribution of the synthesized Fe_3_O_4_@mPEG-Ag hybrid-NPs.

### UV-visible absorption and ROS generation analyses

**Fig. 3.**
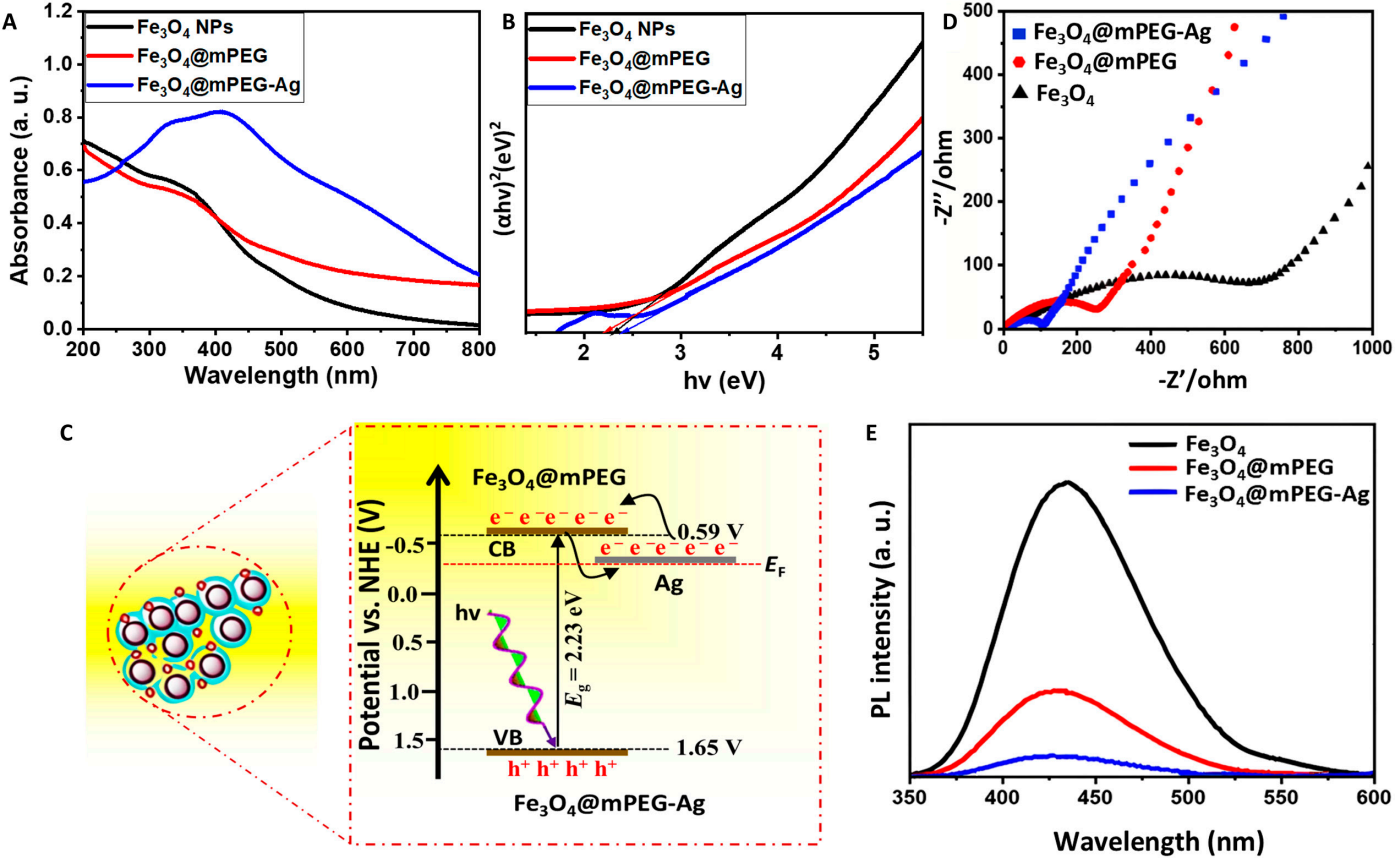
(A) Optical absorption. (B) Energy bandgap Tauc plot. (C) Schematic diagram of the PCC separation and transport mechanism. (D) Nyquist EIS plot. (E) PL spectra of the prepared NPs.

The UV-visible absorbance profile and the corresponding optical bandgaps of the synthesized NPs are displayed in Fig. 3A and B. The absorbance spectra for Fe_3_O_4_, Fe_3_O_4_@mPEG, and Fe_3_O_4_@mPEG-Ag NP samples show characteristic optical absorption bands at varying wavelengths. The pristine Fe_3_O_4_ NP sample exhibits a broad absorption profile predominantly in the UV region with a notable absorption around 380 to 390 nm. This characteristic band corresponds to LMCT (ligand-to-metal charge transfer) transitions in the Fe_3_O_4_ core due to the magnetic features of the magnetite NPs [37]. Beyond this, the absorption tailing into the visible region suggests the intrinsic electronic transitions of Fe ions (Fe^+^), which remain a hallmark of the pristine Fe_3_O_4_ materials [27,37]. The modification with mPEG enhances the optical response of the Fe_3_O_4_@mPEG NP sample, indicating improved light absorption characteristics. The absorption spectrum exhibits a broader tailing into the visible region compared to the pristine Fe_3_O_4_ NPs. This enhancement is attributed to the surface functionalization of Fe_3_O_4_ with mPEG, which improves NP dispersion, reduces aggregation, and minimizes light scattering effects. The presence of mPEG also introduces organic functional groups that contribute to electronic transitions, thereby extending the optical response into the visible region. Furthermore, the integration of Ag NPs onto the Fe_3_O_4_@mPEG further amplifies the optical absorption, particularly in the visible region. A prominent absorption band centered around 430 to 450 nm can be observed, which assigns the LSPR (localized surface plasmon resonance) of the Ag NPs under light exposure [38]. This distinct plasmonic peak significantly enhances the Fe_3_O_4_@mPEG-Ag NP ability to absorb visible light, resulting in superior light-harvesting capabilities across the UV-visible range [31,38]. This progressive enhancement highlights the impact of surface engineering and the integration of plasmonic NPs in improving the optical properties of hybrid nanomaterials, making the Fe_3_O_4_@mPEG-Ag NP system highly suitable for superior photocatalysis in light-harvesting applications. To access its optical characteristics, bandgap values were subsequently calculated using Tauc plots, illustrating a linear correlation between the optical absorption band (α*hν*)*^n^* and photon energy (*hν*), as shown in Fig. 3B [33,39]. Here, *n* corresponds to the type of bandgap, with *n* equal to 2 for a direct transition and ^1^/_2_ for indirect ones, *hν* represents the energy of the incoming photon, and α refers to the material absorption coefficient. Tauc plots analysis revealed bandgap energies of 2.33 eV for Fe_3_O_4_, 2.23 eV for Fe_3_O_4_@mPEG, and 2.45 eV for Fe_3_O_4_@mPEG-Ag NPs, indicating that the mPEG surface modification lowers the bandgap of Fe_3_O_4_. This reduction can be attributed to electronic interactions between Fe_3_O_4_ and mPEG, which introduce surface defect states and modify the local electronic structure. These defect states facilitate additional electronic transitions, which effectively reduce the energy required for excitations. Furthermore, the subsequent incorporation of Ag onto Fe_3_O_4_@mPEG NPs results in an increased bandgap, suggesting the strengthening of electronic interactions and a reduction in charge carrier recombination. This modification indicates the role of Ag NPs in augmenting the charge transfer dynamics between Fe_3_O_4_ and Ag across the mPEG interface, while the larger bandgap ensures that visible light energy remains sufficient for excitation, thereby enabling effective PCC separation. This improved charge carrier transport ultimately resulted in enhanced optical performance, highlighting the synergistic effects of both mPEG surface functionalization and Ag integration in driving the photocatalytic antibacterial performance of Fe_3_O_4_. Figure 3C schematically illustrates the photocatalytic charge transfer mechanism, where Fe_3_O_4_ efficiently absorbs visible light, leading to the transition of excited electrons from VB to CB. The photoexcited electrons are then efficiently transferred across the mPEG interface to the surface-loaded Ag, which possesses a lower Fermi energy level (*E*_F_) relative to the CB edge of Fe_3_O_4_. This interfacial charge migration pathway significantly enhances charge carrier separation, minimizes electron-hole recombination, and thereby optimizes the overall photocatalytic efficiency. To further investigate the charge carrier separation efficiency and interfacial charge transport properties of the synthesized nanomaterials, electrochemical impedance spectroscopy (EIS) measurements were performed. According to Fig. 3D, the Nyquist plot reveals distinct differences across all 3 tested materials. Pristine Fe_3_O_4_ displayed the largest semicircle radius, signifying a higher charge transfer resistance due to the rapid charge recombination. Upon mPEG modification, the Fe_3_O_4_@mPEG sample demonstrated a reduced semicircle radius, suggesting enhanced charge transport facilitated by the surface passivation effect of mPEG. Notably, the Fe_3_O_4_@mPEG-Ag sample exhibited the smallest semicircle radius, signifying the lowest charge transfer resistance. This substantial decrease in impedance is attributed to the efficient electron extraction and release capabilities of the surface-loaded Ag, which acts as both an electron sink and charge mediator, thereby enhancing interfacial charge migration and promoting efficient charge transport. These results further confirm the superior charge transfer dynamic in Fe_3_O_4_@mPEG-Ag NPs, making them a promising candidate for photocatalytic applications. To validate the EIS findings, the photoluminance (PL) test was performed to further evaluate the mobility or reduction in PCC transport. As depicted in Fig. 3E, the Fe_3_O_4_ sample exhibited the highest PL intensity, indicating a swift recombination of PCCs. Upon mPEG functionalization, the PL intensity of Fe_3_O_4_@mPEG was markedly quenched, confirming improved electron/hole pair separation. The Fe_3_O_4_@mPEG-Ag sample displayed the lowest PL intensity among the 3 samples, highlighting the role of Ag in further suppressing the charge recombination through its efficient charge-capturing and releasing potential. These findings, combined with EIS and optical absorption analysis, demonstrate that Fe_3_O_4_@mPEG-Ag hybrid-NPs possess superior charge transport properties, reduced interfacial charge resistance, and enhanced PCC mobility under visible light, highlighting their potential for photodriven eradication of drug-resistant infectious bacteria.

**Fig. 4.**
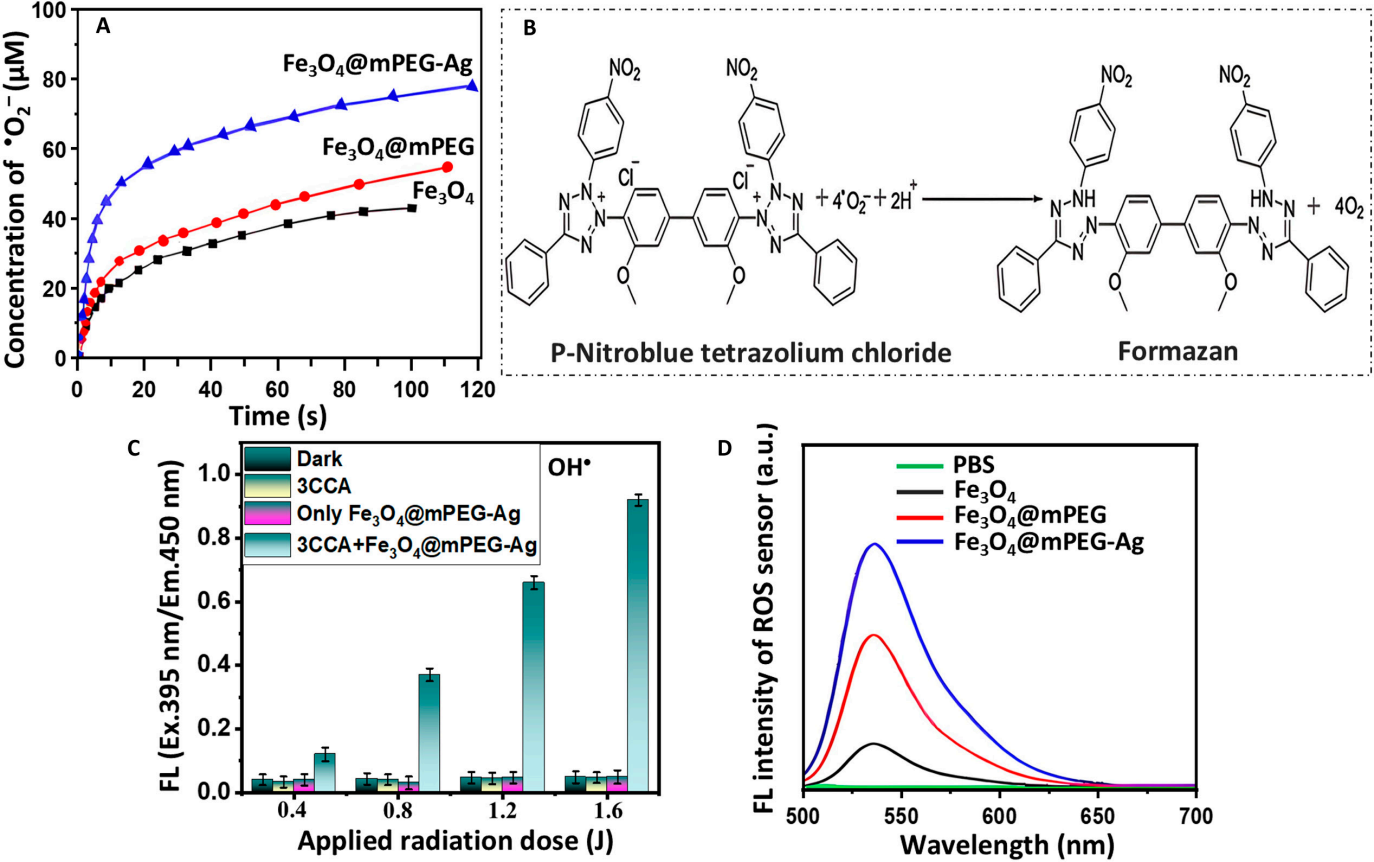
(A) Temporal behavior of superoxide (^•^O_2_^−^) radical quenching. (B) Reduction of p-NBT into formazan. (C) Variation in fluorescence within the ROS sensor system comprising Fe_3_O_4_@mPEG-Ag hybrid-NPs and 3-CCA upon UV-visible light exposure. (D) DCFH-DA fluorescence spectra of the respective Fe_3_O_4_, Fe_3_O_4_@mPEG, and Fe_3_O_4_@mPEG-Ag NP samples.

To evaluate the formation of the active radicals in the developed materials, a series of quenching experiments were performed to identify the main species and/or electrons/holes (e^−^/h^+^) responsible for accelerating the photocatalytic reactions. Specific probe molecules, including p-NBT for superoxide (^•^O_2_^−^) radicals, 3-carboxy coumarin acid (3-CCA) for ^•^OH radicals, and DCFH-DA for overall ROS detection, were employed as chemical scavengers. Their selective reactivity enabled the precise identification of active species generated during photoreactions, offering important insights into the photocatalytic and photodynamic mechanisms of the nanomaterials [40]. Typically, the concentration of generated ^•^O_2_^−^ radicals during the photocatalysis is shown in Fig. 4A. After 120 min of incubation, the Fe_3_O_4_, Fe_3_O_4_@mPEG, and Fe_3_O_4_@mPEG-Ag NPs produced detectable amounts of ^•^O_2_^−^ radicals with concentrations of 43.7, 57.4, and 78.6 μM, respectively. This scavenging activity indicates an enhanced ^•^O_2_^−^ radical generation capability in the Fe_3_O_4_@mPEG-Ag NP sample, highlighting the synergistic effect of the composite structure in boosting the photogenerated reactive species production. The results presented in Fig. 4A are derived from an in-depth study of the interaction between p-NBT and ^•^O_2_^−^ radicals at a strictly maintained molar ratio of 1:4. This critical reaction is visually depicted in Fig. 4B, demonstrating the measurable detection of the ^•^O_2_^−^ radicals using p-NBT as a sensitive probe molecule. These findings provide valuable insights into the identification and behavior of ^•^O_2_^−^ radicals within the photodynamic systems, advancing our knowledge of their generation, structural features, and their essential contribution to photocatalytic processes. To further explore the photochemical reactivity of the synthesized materials, 3-CCA was used as a probe molecule to detect the formation of ^•^OH radicals and photogenerated h^+^. These experiments were carefully controlled using UV-visible radiation from a high-intensity light-emitting diode (LED) source at RT. The interaction between ^•^OH radicals and 3-CCA under light exposure yielded 7-hydroxycourmarin-3-carboxylic acid, which exhibits a distinct fluorescent signal. This fluorescence emission served as a clear indication of ^•^OH radicals’ generation, highlighting the NPs’ potential for efficient photochemical activities. As shown in Fig. 4C, UV-visible illumination of the reaction mixture contained Fe_3_O_4_@mPEG-Ag hybrid-NPs and 3-CCA, leading to the considerable formation of 7-hydroxycourmarin-3-carboxylic acid, displaying pronounced fluorescent properties. The increase in the fluorescence intensity with elevated radiation doses indicates the formation of ^•^OH radical during the photochemical process. The boosted signal response is primarily attributed to the surface hydroxyl groups in the Fe_3_O_4_ component of the hybrid-NPs, which play a crucial role in facilitating ^•^OH radical formation and contributing to the NP system photodynamic activity. In contrast, no significant alteration in the fluorescence intensity was observed in the non-illuminated reaction or irradiated individual component (Fe_3_O_4_@mPEG-Ag hybrid-NPs or 3-CCA). This indicates that the formation of 7-hydroxycourmarin-3-carboxylic acid occurs due to the photocatalytic interaction between hybrid-NPs and 3-CCA upon UV-visible irradiation, confirming the necessity of both components and light activation for generating ^•^OH radicals. In the reaction system, chloride ions (Cl^−^) play a key role in effectively capturing photogenerated h^+^, thereby indirectly promoting the oxidation of 3-CCA molecules [41]. This process enhances the generation of reactive intermediates, contributing to the observed oxidation of 3-CCA and the formation of the fluorescent 7-hydroxycourmarin-3-carboxylic acid, further supporting the proposed photocatalytic mechanism. Additionally, an evaluation of the complete ROS production potential of the synthesized NPs was performed by analyzing fluorescence signals, as depicted in Fig. 4D. The Fe_3_O_4_@mPEG-Ag hybrid-NPs displayed potentially stronger fluorescence under UV-visible irradiation with DCFH-DA, compared to Fe_3_O_4_ and Fe_3_O_4_@mPEG NP samples. This enhanced fluorescence indicates that the Fe_3_O_4_@mPEG-Ag hybrid-NPs generate a greater degree of ROS, indicating their superior capability of ROS production and reinforcing the sample potential for strong photodynamic activities. Additionally, the ESR analysis (Fig. S2A and B) further confirmed that all 3 samples produced both ^•^O_2_^−^ and ^•^OH radicals following 20 min of exposure to visible light. Comparative analysis revealed that the Fe_3_O_4_@mPEG-Ag hybrid-NP sample generated markedly higher levels of these reactive species than both Fe_3_O_4_ and Fe_3_O_4_@mPEG NPs. This enhanced radical generation accentuates the superior photodynamic performance of the resulting hybrid-NP system, indicating their strong potential for effective photodynamic antibacterial action against multiple drug-resistant microbes.

### Visible light-induced bacterial eradication

**Fig. 5.**
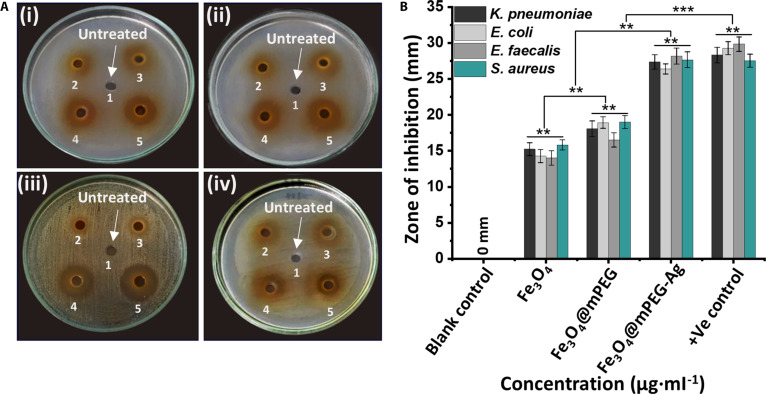
(A) Antibacterial performance of the developed materials and control groups (1 signifies blank control–0 μg·ml^−1^ and 5 is the positive control group corresponding to CIP treatment at 25 μg·ml^−1^) targeting (i) *K. pneumoniae*, (ii) *E. coli*, (iii) *E. faecalis*, and (iv) *S. aureus* bacterial strains under visible light treatment conditions. (B) Bar diagram illustrating the corresponding inhibition zone diameters (mm) of treated pathogens across different NP dosages: 0 μg·ml^−1^ (Fe_3_O_4_@mPEG-Ag NPs), 50 μg·ml^−1^ (Fe_3_O_4_@mPEG-Ag NPs), 100 μg·ml^−1^ (Fe_3_O_4_@mPEG NPs), 150 μg·ml^−1^ (Fe_3_O_4_ NPs), and 25 μg·ml^−1^ (CIP). A direct comparison was made initially between each antibacterial agent and bacteria like *K. pneumoniae* at equal dosages, followed by a cross-analysis among all 4 formulations at various doses. Significance levels were indicated by *P < 0.05, **P < 0.01, and ***P < 0.001.

The antibacterial performance of the synthesized Fe_3_O_4_, Fe_3_O_4_@mPEG, and Fe_3_O_4_@mPEG-Ag NPs was evaluated on 2 types of drug-resistant bacteria: Gram-negative strains (*K. pneumoniae, E. coli*) and Gram-positive strains (*E. faecalis, S. aureus*), under visible light irradiation and in dark conditions using the standard agar well diffusion protocol. Their antibacterial effectiveness was determined by measuring the diameter of the growth inhibition zone in millimeters across different concentrations, as depicted in Fig. 5A and B. The obtained results were compared with the reference standard values established by CLSI [33] as presented in Table 1. The corresponding inhibition zone data in Fig. 5A illustrate the bactericidal performance of the evaluated material samples against each bacterial strain under visible light and are further quantified through ZOI measurements, as illustrated in the bar graph of Fig. 5B. A comparison with the CLSI reference zone diameters revealed that the Fe_3_O_4_@mPEG-Ag hybrid-NP sample possesses robust antibacterial properties against all 4 targeted pathogens. Typically, the bactericidal capability of the synthesized NPs was systematically examined across the 0 to 150 μg·ml^−1^ concentration spectrum. At 0 μg·ml^−1^, bacterial growth remained entirely unimpeded, as indicated by the absence of any inhibition zones. This outcome confirms that all 4 bacterial strains proliferated freely. However, introducing the antibacterial NPs led to the formation of noticeable inhibition zones, signifying their effectiveness in suppressing bacterial growth. At an elevated concentration of Fe_3_O_4_ (75 μl of 150 μg·ml^−1^) and Fe_3_O_4_@mPEG (75 μl of 100 μg·ml^−1^), the bacterial strains *K. pneumoniae*, *E. coli*, *E. faecalis*, and *S. aureus* exhibited intermediate inhibition zone diameters of (15.21 ± 0.9 mm), (14.24 ± 0.8 mm), (14.05 ± 1.0 mm), and (15.80 ± 0.7 mm) for pristine Fe_3_O_4_, and (18.04 ± 1.1 mm), (18.90 ± 0.7 mm), (16.52 ± 1.0 mm), and (18.97 ± 0.9 mm) for Fe_3_O_4_@mPEG under visible light irradiation, respectively. In contrast, the Fe_3_O_4_@mPEG-Ag hybrid-NP sample, even at a reduced (75 μl of 50 μg·ml^−1^) concentration exhibited larger inhibition zones of (27.35 ± 1.0 mm), (26.42 ± 0.7 mm), (28.15 ± 1.2 mm), and (27.64 ± 1.1 mm) under 1.6-W dose of visible light irradiation. The positive control group (CIP) exhibited substantial inhibition zones toward *K. pneumoniae* (28.25 ± 1.1 mm), *E. coli* (29.26 ± 0.9 mm), *E. faecalis* (29.81 ± 1.0 mm), and *S. aureus* (27.52 ± 0.8 mm) at 75 μl of 25 μg·ml^−1^. Following the preliminary antibacterial assessment, the resulting hybrid-NPs exhibited encouragingly greater ZOIs compared to both the blank control and counterpart samples and demonstrated almost equal antibacterial performance to that observed for the reference antibiotic CIP. The respective inhibition zone diameters observed for the Fe_3_O_4_@mPEG-Ag hybrid-NP sample targeting all tested strains exceeded the CLSI guidelines, further confirming the strong antibacterial activity of the resultant hybrid-NPs. In addition, the bacterial growth curve assay further confirmed these findings, illustrating that all 4 bacterial strains treated with Fe_3_O_4_@mPEG-Ag hybrid-NPs under visible light exhibited no bacterial proliferation, while untreated bacteria entered a rapid logarithmic growth phase, beginning shortly after a brief lag phase, and attained a steady state within 9 to 10 h (Fig. S3A to D). A thorough analysis reveals that the Fe_3_O_4_@mPEG-Ag hybrid-NPs exhibit notable inhibitory biocidal efficacy against both types of pathogenic strains (Gram-positive and Gram-negative), even at a relatively low dosage (50 μg·ml^−1^). This performance is far superior to those of its counterparts under both illuminated and non-illuminated (see the details of ESI; Fig. S4) conditions, with efficacy similar to the CIP. The enhanced antibacterial activity is mainly due to the robust electrostatic interaction between the NPs and bacterial membranes. Additionally, the strong photocatalytic response of the hybrid-NPs under light effectively separates e^−^/h^+^ pairs, ultimately enhancing their photodynamic antimicrobial capabilities.

**Table 1.** Reference zone of inhibition according to CLSI guidelines

Antibacterial agent	Measured zone of inhibition in mm		
	Vulnerable	Intermediate	Resilient
Fe_3_O_4_@mPEG-Ag	>19	14–19	<14

**Fig. 6.**
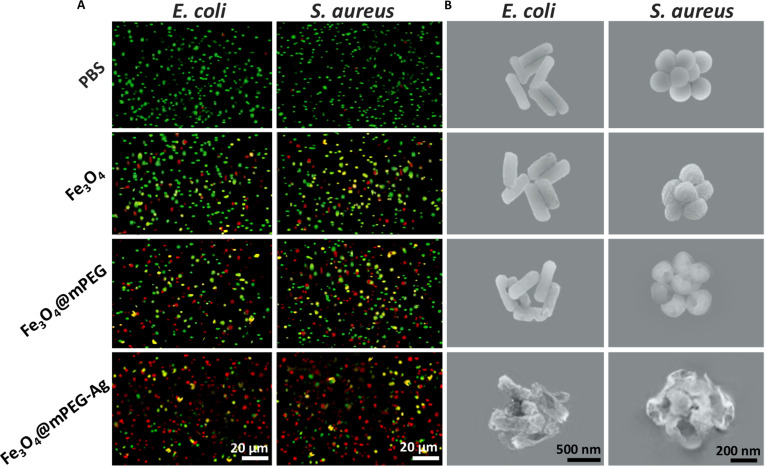
(A) Fluorescence images of live/dead pathogens treated with PBS, Fe_3_O_4_, Fe_3_O_4_@mPEG, and Fe_3_O_4_@mPEG-Ag hybrid-NPs under visible light irradiation. (B) SEM micrographs of the PBS, Fe_3_O_4_, Fe_3_O_4_@mPEG, and Fe_3_O_4_@mPEG-Ag hybrid-NP-treated pathogens under visible light irradiation.

To gain deeper insights into the biocidal effectiveness of the synthesized materials, the structural integrity of the NP-treated both Gram-positive (*S. aureus*) and Gram-negative (*E. coli*) cells was assessed through advanced laser-based confocal imaging, as displayed in Fig. 6A. A double staining approach was applied using SYTO 9 and propidium iodide (PI) as 2 fluorescent acid dyes to distinguish between live and dead cells. SYTO 9 dye produces a green signal by penetrating the bacterial cytoplasmic membrane and binding to nucleic acids in both live and dead cells, while PI produces red fluorescence and exclusively marks dead cells due to its cell-impermeable features [42]. As shown in Fig. 6A, *E. coli* and *S. aureus* treated with PBS (control group) exhibited noticeable green fluorescence, indicating a high proportion of live bacterial cells, while only a few bacterial cells displayed red fluorescence, representing minimal cell membrane damage. In contrast, some red fluorescence appeared in the Fe_3_O_4_ and Fe_3_O_4_@mPEG NP-treated groups, with the red signal becoming gradually more intense in the case of the Fe_3_O_4_@mPEG-treated sample, reflecting the synergistic eradication effect of Fe_3_O_4_ and mPEG in the sample. Interestingly, bacterial cells exhibited stronger red fluorescence after being treated with the Fe_3_O_4_@mPEG-Ag hybrid-NP group, indicating strong visible light-activated bactericidal effects against both strains, aligning with the previously reported antimicrobial findings [25–27,43,44]. Notably, the confocal images of NP-treated *E. coli* displayed a kind of rounded morphology, which was not due to the structural deformation or cell contamination. Instead, it might be caused by optical imaging artefacts related to fluorescence projection, *z*-axis compression, and signal overlap during 3D image acquisition. This interpretation is supported by prior reports where *E. coli* cells appeared as rounded spots under confocal microscopy due to limited axial resolution and fluorophore distribution, despite maintaining their rod-shaped morphology under an electron microscope [45,46]. To further confirm their destructive nature, scanning electron microscopy (SEM) analysis was conducted to examine the bacterial cell surface morphology, as illustrated in Fig. 6B. It can be seen that the PBS-treated *E. coli* and *S. aureus* samples displayed intact and smooth cell morphology, manifesting negligible cell membrane damage. In contrast, the SEM micrographs of the bacterial cells show wrinkled, broken, aggregated, and damaged morphology after being treated with Fe_3_O_4_, Fe_3_O_4_@mPEG, and Fe_3_O_4_@mPEG-Ag hybrid-NPs, respectively. The considerable damage to bacterial cells is mainly attributed to the electrostatic interactions of the bactericidal NPs with bacterial cell membranes, which enhance ROS generation under visible light irradiation. This process induces oxidative damage to critical cellular components. Furthermore, the piercing action of the hybrid-NPs inflicts physical damage on the cytoplasmic structure and disrupts cellular contents, ultimately leading to bacterial cell death. These results suggest that the energetic adherence of Fe_3_O_4_@mPEG-Ag NPs to pathogenic cell walls significantly increases their vulnerability to photodynamic action, thereby leading to light-induced cell death.

**Fig. 7.**
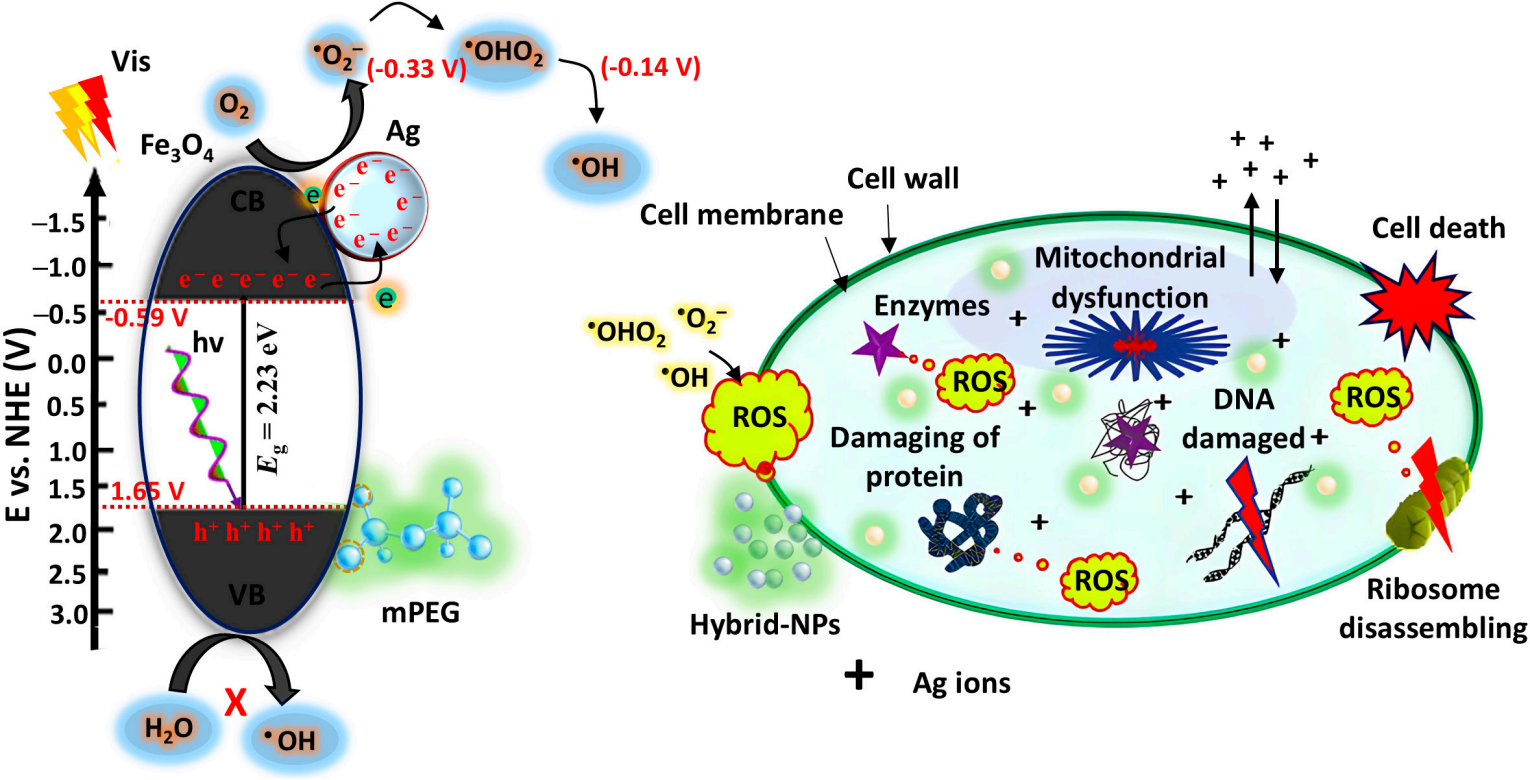
Illustrative representation of the proposed charge transfer pathway and associated reactions, highlighting the antibacterial mechanism of the Fe_3_O_4_@mPEG-Ag hybrid-NPs under visible light irradiation.

Beyond their broad-spectrum antibacterial properties, we aimed to investigate the photocatalytic mechanism of Fe_3_O_4_@mPEG-Ag hybrid-NPs. As a highly efficient semiconductor photocatalyst, these hybrid-NPs generate high levels of reactive species under visible light irradiation, which play a crucial role in inactivating targeted bacteria. Under visible light illumination, these hybrid-NPs lead to the generation of e^−^/h^+^ pairs. These photoexcited e^−^/h^+^ pairs participate in various reactions that ultimately result in the production of cytotoxic ROS, such as ^•^O_2_^−^ and ^•^OH radicals through the reaction pathway of $O_{2}\to O_{2}^{•-}$ ↔OH^•^ [40,47]. These reactive species cause oxidative harm to essential biomolecules in pathogens by initially disrupting their outer structure, leading to the release of intracellular contents and ultimately resulting in cell lysis (Fig. 7). The identification of key antibacterial mechanisms responsible for inhibiting bacterial growth is crucial for developing effective strategies against drug-resistant pathogens, particularly through the use of nanomaterials [33,48–50]. A key antibacterial strategy involves the direct contact of these nano-materials with pathogenic cell membranes, causing structural damage and release of internal materials. This effectively suppresses bacterial growth. Optimizing the size and specific surface area of these nanomaterials can further enhance their biocidal potential [51,52]. Another key approach involves the control releasing of metal ions into the bacterial milieu, inducing oxidative and enzymatic stress that disrupts cell membranes. Fine-tuning the release of metal ions and considering the particle structure, along with a specific type of targeted microorganism, can further optimize their therapeutic impact [51,53]. Furthermore, the exposure of nanomaterials to light can generate cytotoxic ROS, which directly interact with bacterial contents. The interaction of hybrid-NPs with bacterial cells causes oxidative stress, disrupting vital cellular structures such as lipids, proteins, and DNA. This disruption effectively inhibits the growth and proliferation of the pathogens [53,54]. In this aspect, the photocatalytic mechanism of the developed Fe_3_O_4_@mPEG-Ag hybrid-NPs upon light exposure was pointedly augmented due to the combined mPEG coating and Ag loading-induced effects. These modifications contribute to improved PCC separation and enhanced absorption efficiency. To further elucidate the electronic properties of the hybrid-NPs, Mullikan’s electronegativity theory was applied to determine the redox potentials of CB and VB [55]. This approach yields valuable insights into the electronic characteristics and operational effectiveness of the nanomaterials in photocatalytic applications.

$$E_{\left(\mathrm{VB}\right)}=X–E_{\left(C\right)}+\;\frac{1}{2}E_{\left(g\right)}$$ (1)

$$E_{\left(\mathrm{CB}\right)}=E_{\left(\mathrm{VB}\right)}–E_{\left(g\right)}$$ (2)

Here, *X* refers to the average electronegativity of atoms, and for Fe_3_O_4_, this value is nearly 5.03 eV, and *E*_(C)_ represents the conversion constant (4.50 eV) that links the vacuum energy scale to the NHE (normal hydrogen electrode) scale. The estimated energy levels for VB and CB are 1.65 V and −0.59 V, respectively. This suggests that the CB electrons possess sufficiently low potential to engage in redox reactions relative to oxygen reduction potential in water (e.g., E° O_2_/^•^O_2_^−^ = −0.33 V and E° O_2_/^•^HO_2_ = −0.13 V versus NHE) [50,56]. These values correspond well with the electronic configuration of the hybrid-NPs. From these results, the band structure of Fe_3_O_4_@mPEG [*E*_(VB)_ = 1.65 eV, *E*_(CB)_ = −0.57 eV, and *E*_(g)_ = 2.23 eV] combined with the surface-loaded Ag NPs effectively enhances the generation and mobility of charge carriers when subjected to visible light illumination (Fig. 7). The improved separation and transport of PCCs facilitates the efficient conversion of adsorbed oxygen species into ROS on the photocatalyst surface. By comparing with the prior ESR analysis, it can be inferred that the CB is responsible for forming highly reactive oxygen intermediates, such as ^•^O_2_^−^ and ^•^HO via reduction reactions outlined in Eqs. (3) to (7). On the other hand, VB-h^+^ lacks the energy needed to directly oxidize aqueous solution to ^•^OH species via a conventional oxidation route [shown in Eq. (8)]. This is because the VB level of Fe_3_O_4_@mPEG lies below the oxidation potential of H_2_O/^•^OH (2.37 eV versus NHE) [50,57]. As a consequence, the photo-generated h^+^ can function as a direct potent agent in the antibacterial performance of the NP system. Moreover, the presence of mPEG enhances the trapping of VB-h^+^ owing to the high density of amine functionalities, thereby promoting PCC separation and intensifying ROS formation. These observations are consistent with the data obtained from active species quenching tests and ESR spectra active species trapping assay and ESR analysis, thereby confirming the generation of ROS (^•^O_2_^−^, ^•^OH) and h^+^ in the Fe_3_O_4_@mPEG-Ag hybrid-NP system. Furthermore, it is noteworthy that aerobic microbes inherently produce substantial amounts of free-radicals during their metabolic activities [refer to Eqs. (9) and (12)] [58]. In a normal condition, pathogenic cells possess intrinsic molecules that neutralize these radicals by forming stable compounds, which are essential for preserving mitochondrial respiratory chain function [40,59]. However, prolonged exposure to externally introduced ROS can induce a notable oxidative stress, disrupting this balance. Persistent oxidative pressure can impair the enzymatic system of pathogens, ultimately diminishing their physiological function [50,59,60]. To summarize the discussion, the as-synthesized Fe_3_O_4_@mPEG-Ag hybrid-NP photocatalyst can effectively anchor to bacterial cell membranes through electrostatic interactions due to the cationic nature of the mPEG [as shown in Eq. (11)], while the Ag component enhances this interaction by disrupting the membrane integrity and exhibiting bactericidal activity through controlled Ag^+^ release (Fig. S5) and ROS generation. In short, upon visible light irradiation, these hybrid-NPs generate a substantial quantity of ROS (^•^O_2_^−^, ^•^OH) radicals, and h^+^ through efficient electron transfer from Ag to Fe_3_O_4_ across the mPEG interface [as described in Eq. (12)]. Additionally, a regulated leakage of Ag^+^ was ensured through an ICP-OES test (see the ESI for details). Overall, these processes trigger intense oxidative pressure and compromise the structural integrity of pathogenic cell walls, thereby contributing to the eradication of drug-resistant bacterial strains.

The generation of active species in the synthesized materials was additionally validated through the evaluation of total protein content and CAT activity, as shown in Fig. S6A to D. In Fig. S6A and B, a reduction in protein levels is observed for both tested pathogenic strains compared to the blank control sample, highlighting the ability of hybrid-NPs to suppress protein synthesis. In addition, Fig. S6C and D illustrates a noticeable increase in CAT activity in bacterial species exposed to hybrid-NPs compared to blank control groups, reflecting strong bactericidal efficacy through enhanced ROS production and membrane disruption. In contrast to the blank control group, the resultant hybrid-NPs exhibit remarkably higher protein leakage

$$\begin{array}{c} {\mathrm{Fe}}_{3}O_{4}@\text{mPEG}-\mathrm{Ag}+\mathrm{hv}↔{\mathrm{Fe}}_{3}O_{4}@\text{mPEG}\\ -{\mathrm{Ag}}^{⋆}+\left({h^{+}}_{\mathrm{VB}}\right)+\left({e^{-}}_{\mathrm{CB}}\right) \end{array}$$ (3)

$$\mathrm{Ag}+\mathrm{hv}\to {\mathrm{Ag}}^{⋆}+{e^{-}}_{\mathrm{hot}}$$ (4)

$${\mathrm{Ag}}^{⋆}\left({e^{-}}_{\mathrm{hot}}\right)+{{\mathrm{Fe}}_{3}O_{4}}^{⋆}\to {\mathrm{Fe}}_{3}O_{4}\left({e^{-}}_{\mathrm{CB}}\right)$$ (5)

$${\mathrm{Fe}}_{3}O_{4}@\text{mPEG}-{\mathrm{Ag}}^{⋆}\left({e^{-}}_{\mathrm{CB}}\right)+O_{2}\to O_{2}^{•-}$$ (6)

$$O_{2}^{•-}+2H^{+}\to {\;}^{•}{\mathrm{HO}}_{2}\to {\mathrm{OH}}^{•}\;$$ (7)

$${\mathrm{Fe}}_{3}O_{4}@\text{mPEG}\left({h^{+}}_{\mathrm{VB}}\right)⋆+H_{2}O/{\mathrm{OH}}^{-}\to {\mathrm{OH}}^{•}$$ (8)

$$O_{2}+e\to O_{2}^{•-}$$ (9)

$$H_{2}O_{2}+O_{2}^{•-}\to O_{2}+{\mathrm{OH}}^{-}+{\mathrm{OH}}^{•}$$ (10)

$$\begin{array}{c} \text{mPEG}-{\mathrm{Ag}}^{+}+\text{Bacterial membrane}\to \\ \text{Tight surface adherence induced cell disruption}\; \end{array}$$ (11)

$$\begin{array}{c} O_{2}^{•-},{\mathrm{OH}}^{•},\;h^{+}\to \to \to \text{Active radical}\\ -\text{based antipathogenic activities} \end{array}$$ (12)

and CAT activity, nearly comparable to the positive control group (standard antibiotic CIP), confirming their strong bioactivity. These observed variations in both protein content and CAT activity assays in respective Fe_3_O_4_@mPEG and Fe_3_O_4_@mPEG-Ag hybrid-NP-treated samples affirm the generation of active radicals, providing compelling evidence for the formation of highly reactive intracellular reactive species.

Based on the aforementioned experimental analysis, the as-synthesized materials demonstrated remarkable antibacterial activity by effectively targeting pathogenic strains and disrupting their cellular structures through direct membrane interaction. The elevated generation of reactive intermediate species (RIS), including oxygen and metal ions, intensified oxidative stress, ultimately leading to bacterial cell death. Compared to earlier reported bactericidal agents, the Fe_3_O_4_@mPEG-Ag hybrid-NPs exhibited exceptional efficacy even at minimal concentrations (as shown in Table S2), which not only reduces the required dosage but also minimizes potential cytotoxicity and off-target effects toward mammalian cells. While the experimental data confirmed potent antibacterial efficacy primarily induced by RIS damage, it is still necessary to explore possible molecular-level interactions that can further elucidate the bactericidal mechanism. Despite numerous studies that have examined the antibacterial efficacy of metal-based nanomaterials, the precise molecular mechanisms underlying their bioactivity remain incompletely understood [40,61,62]. Computational techniques, particularly molecular docking (in silico), have recently emerged as powerful tools to investigate such interactions, helping experimental findings to predict how nanomaterials engage with biomolecular targets. These insights facilitate the rational design of advanced antimicrobial platforms that target bacterial membranes and enzymes, which are essential for bacterial metabolism and serve as critical targets for developing novel antibacterial agents [63–65].

### Computational validation of the antibacterial mechanism

**Fig. 8.**
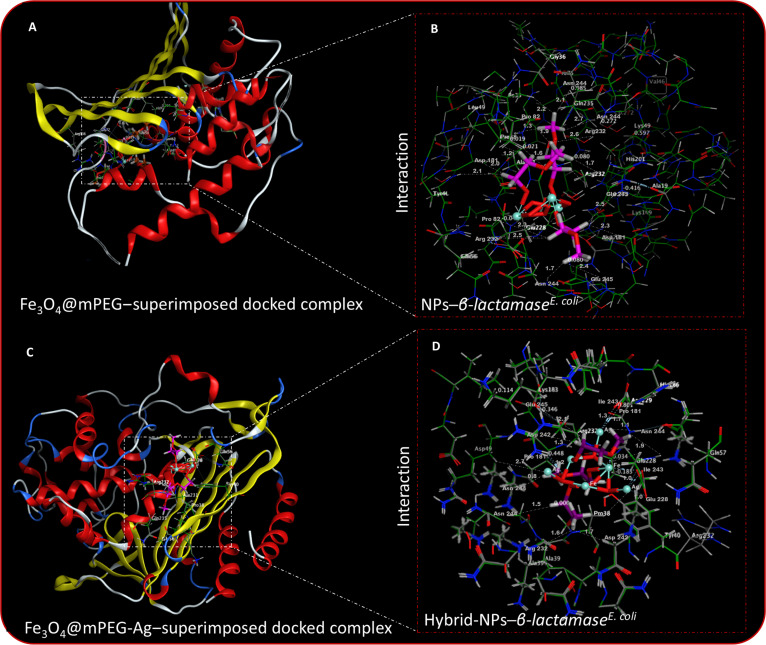
(A and C) NP–*β-lactamase^(E. coli)^* superimposed docking conformation. (B) Fe_3_O_4_@mPEG NP–*β-lactamase^(E. coli)^* complex. (D) Fe_3_O_4_@mPEG-Ag hybrid-NP–*β-lactamase^(E. coli)^* complex.

**Fig. 9.**
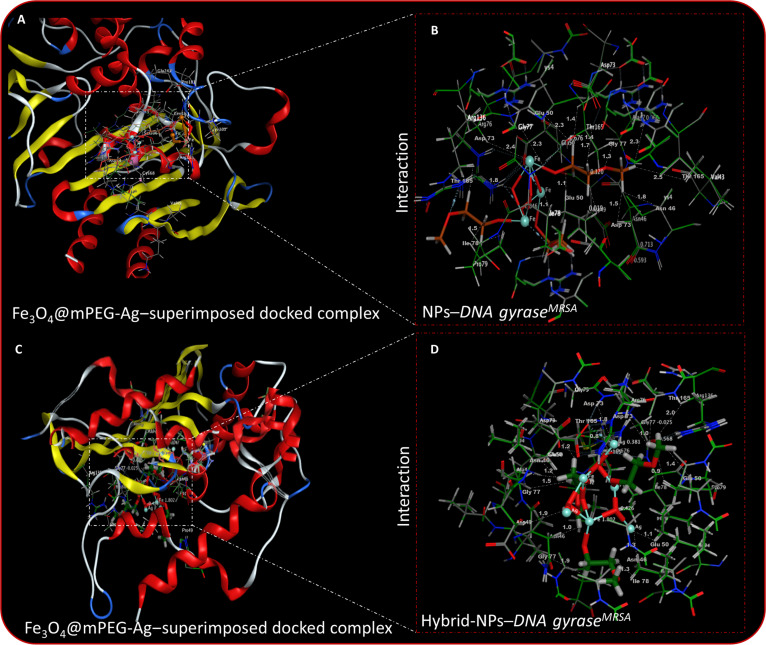
(A and C) NP–*DNA gyrase^(S. aureus)^* superimposed docked complexes. (B) Fe_3_O_4_@mPEG NP–*DNA gyrase^(S. aureus)^* complex. (D) Fe_3_O_4_@mPEG-Ag hybrid-NP–*DNA gyrase^(S. aureus)^* complex.

Molecular docking simulations (in silico) were therefore employed to investigate the interaction between the prepared nanomaterials and key bacterial enzymes involved in drug resistance and viability. By analyzing binding affinities and docking interactions at the molecular level, these studies provide insight into the potential antibacterial mechanisms mediated by interaction with essential bacterial enzymes. This computational approach helps in identifying critical binding sites, evaluating the stability of the NP–enzyme complexes, and predicting their effectiveness in disrupting bacterial growth, thereby supporting the experimentally observed RIS-induced cytotoxicity. Previously reported studies have identified enzymes such as *β-lactamase* and *DNA gyrase* as potentially valuable targets in designing next-generation antibacterial agents, particularly against drug-resistant bacterial strains [66,67]. The present investigations explore the interaction potential of Fe_3_O_4_@mPEG and Fe_3_O_4_@mPEG-Ag hybrid-NPs toward the catalytic domains of *β-lactamase* and *DNA gyrase*, derived from the respective strains of *E. coli* and *S. aureus*. A 3D structural representation between the NPs and *DNA gyrase*, structurally aligned and detailed in Fig. S7A to C, was constructed to illustrate these interactions between the NPs and the functional domains of *DNA gyrase*. These interactions suggest a high binding affinity, potentially disrupting enzymatic activity and contributing to bacterial inhibition. The structural visualizations provide deeper insights into the molecular recognition process, highlighting key interaction sites that may enhance the antibacterial efficacy of the NPs. The Fe_3_O_4_@mPEG NPs exhibited an optimal docking conformation within the active domains of *β-lactamase*, displaying hydrogen-bonding interactions with Pro^181^ (1.3 and 1.6 Å), Glu^245^ (2.4 Å), Asp^242^ (2.0 and 2.3 Å), Asn^244^ (1.7, 2.1, and 2.7 Å), and Arg^232^ (1.7 and 2.5 Å), resulting in a calculated binding energy of −11.164 kcal·mol^−1^. In comparison, the Fe_3_O_4_@mPEG-Ag hybrid-NPs showed enhanced binding affinity through H-bonding interactions with Pro^181^ (1.2 and 1.7 Å), Glu^245^ (2.1 Å), Asp^242^ (1.3 and 1.7 Å), Asn^244^ (1.1 and 1.5 Å), and Arg^232^ (1.6 Å). Additionally, these hybrid-NPs established C–H bond contacts with Ile^243^ (1.0 and 1.3 Å) and Glu^228^ (1.0 and 1.9 Å), resulting in a superior overall binding energy of −19.837 kcal·mol^−1^. These corresponding binding interactions between *β-lactamase* and Fe_3_O_4_@mPEG-Ag hybrid-NP samples are shown in Fig. 8C and D. Similarly, the top-ranked docking conformation of the Fe_3_O_4_@mPEG NPs with the active sites of *DNA gyrase* (Fig. 9A and B) exhibited hydrogen-bonding interactions with Thr^165^ (1.7, 1.8, and 2.5 Å), Asp^73^ (1.5 and 2.4 Å), and Gly^77^ (1.3 and 2.3 Å), along with additional C–H bond exchanges with Asn^46^ (1.8 Å), Glu^50^ (1.1 and 2.3 Å), and Ile^78^ (1.1 and 1.5 Å). These interactions collectively resulted in a total binding energy of −9.742 kcal·mol^−1^. In contrast, the Fe_3_O_4_@mPEG-Ag hybrid-NP sample established comparable H and C–H bonds with Asp^73^ (1.2 and 1.8 Å), Thr^165^ (0.9 and 1.7 Å), Ans^46^ (1.0 and 1.2 Å), Gly^77^ (1.0 and 1.5 Å), Glu^50^ (1.1 and 1.4 Å), and Ile^78^ (0.9 and 1.3Å), representing superior binding strength with a total binding energy of −13.469 kcal·mol^−1^, as depicted in Fig. 9C and D. Figure 9D further highlights the role of specific amino acid residues, such as Asp^73^ (1.3 and 0.5 Å), Gly^77^ (1.0 and 1.3 Å), and Thr^165^ (0.9 and 2.0 Å), in mediating H-bonding interactions. Notably, the integration of Ag into the Fe_3_O_4_@mPEG framework markedly enhanced the binding affinity of the hybrid-NPs toward both *β-lactamase* and *DNA gyrase*, as evidenced by the increased docking interaction scores (more negative binding energies). This enhancement emphasizes the critical contribution of Ag atoms, which provide additional reactive electron-dense surface sites that strengthen coordination with enzymatic residues via H-bonding and electrostatic interactions. Beyond molecular recognition, Ag also promotes plasmonic excitation under visible light irradiation, thereby accelerating RIS-mediated bactericidal processes. These findings establish a clear mechanistic correlation between the in silico docking results and the experimentally observed in vitro antibacterial efficacy, demonstrating that Ag incorporation synergistically elevates both enzyme inhibition and RIS-induced damage. Consequently, these results highlight the potential of Fe_3_O_4_@mPEG-Ag hybrid-NPs as an innovative non-antibiotic nanoplatform for combating drug-resistant microbial infections.

### Evaluation of hemolytic activity and cytocompatibility

**Fig. 10.**
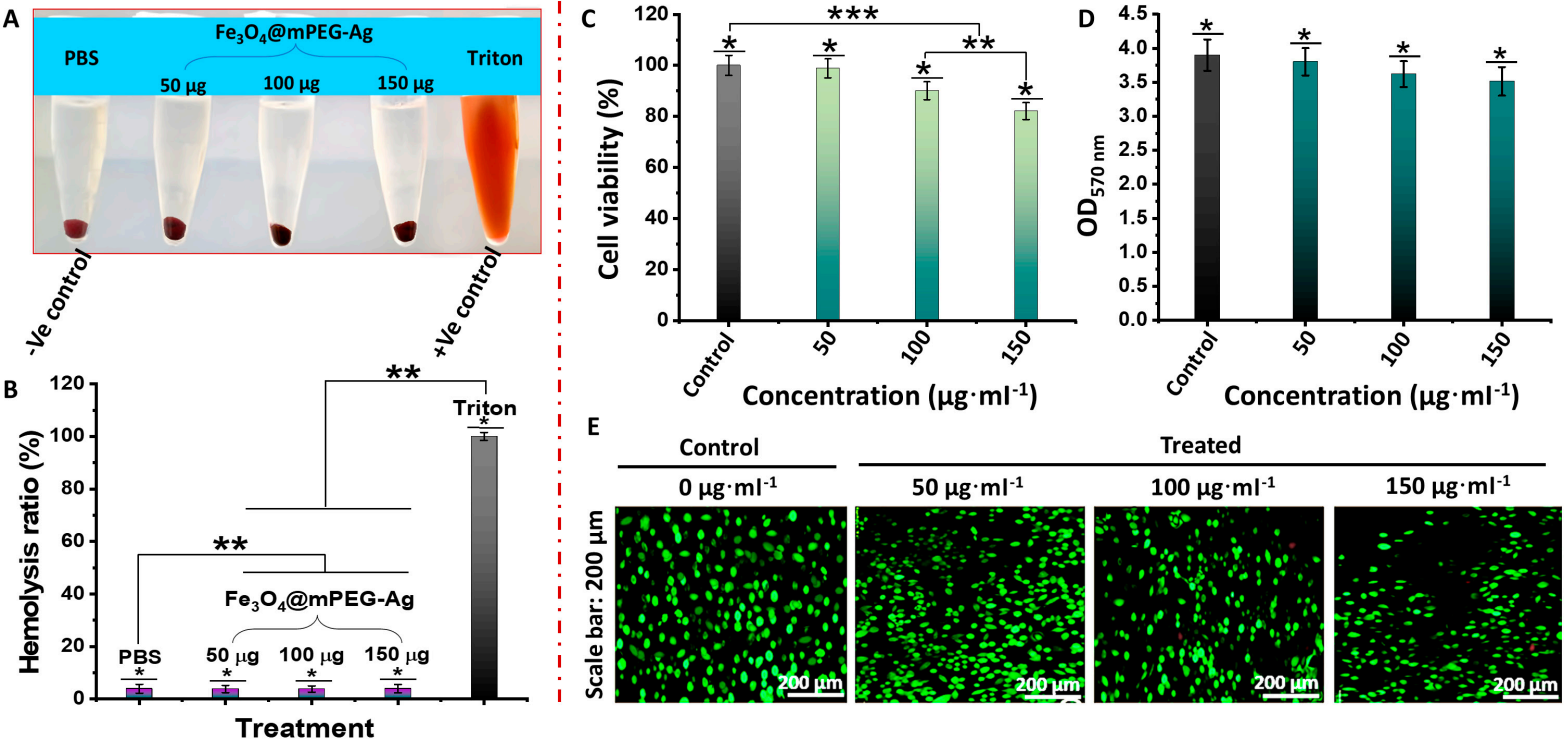
(A) Representative images of hemolytic activity using PBS and Triton X-100 as a respective negative and positive control group, and Fe_3_O_4_@mPEG-Ag NPs at various dosages of 50 to 150 μg·ml^−1^. (B) Hemolysis level of Fe_3_O_4_@mPEG-Ag across tested doses of 50 to 150 μg. Cytotoxicity evaluation: (C) Cell viability percent, (D) OD at 570 nm indicating formazan intensity proportional to viable cells, and (E) fluorescence micrographs of the L929 cells cultured with hybrid-NPs at different concentrations for 24 h.

Lastly, the hemolytic and cytocompatibility profiles of the resulting Fe_3_O_4_@mPEG-Ag hybrid-NPs were comprehensively assessed to ensure minimal adverse effects on mammalian cells. The hemocompatibility assay demonstrated that Triton X-100 was used as a positive control and induced complete hemolysis, which is evident from the intense red colorization of the supernatant. In contrast, RBCs exposed to hybrid-NPs across a concentration range of 50 to 150 μg·ml^−1^ retained their structural integrity, as indicated by a clear, colorless supernatant, similar to the PBS-treated negative control group (Fig. 10A). The quantitative measurements of optical density (OD at 545 nm) (Fig. 10B) confirmed that the hybrid-NP-treated samples demonstrated markedly lower absorbance values than the Triton X-100 group, indicating minimal cell membrane disruption. Hemolysis levels across all tested concentrations remained well below the 5% safety threshold, verifying their biocompatibility with the RBC-contacting environment. Additionally, the biosafety of the hybrid-NPs was further confirmed by evaluating their cytotoxic effects on L929 mouse fibroblast cells, isolated from subcutaneous connective and fat tissues of C3H/An mice. The assessment employed both MTT assays and fluorescence-based staining protocol to evaluate cell viability (Fig. 10C to E). Figure 10C shows the percentage viability of L929 cells treated with varying concentrations (0 to 150 μg·ml^−1^) of the Fe_3_O_4_@mPEG-Ag hybrid-NPs. At 50 μg·ml^−1^ concentration, cell viability was almost like the control group, exceeding 90% at 100 μg·ml^−1^, and even at the highest tested dose of 150 μg·ml^−1^, the cell viability remained over 80%. These results demonstrate the excellent biocompatibility of the Fe_3_O_4_@mPEG-Ag hybrid-NPs across the tested concentrations. This sustained cell viability across increasing concentrations suggests minimal cytotoxicity, which is 3 times higher than the originally designated dose used for the eradication of drug-resistant bacteria. These results were further validated by OD measurements at 570 nm (Fig. 10D), which showed a negligible decrease in OD values with increasing concentrations of the antibacterial compound, indicating excellent cytocompatibility even at elevated doses. Finally, fluorescence staining of the cells in Fig. 10E reveals well-defined cell morphology across all tested concentrations, further ensuring a remarkable in vitro biocompatibility of the hybrid-NPs. Overall, these findings confirm that the developed antibacterial agents induce negligible hemotoxicity and exhibit low cytotoxicity toward the L929 fibroblast cell line, thereby emphasizing their strong biocompatibility for biomedical practices, particularly in scenarios involving prolonged exposure with minimal adverse effects.

In summary, the Fe_3_O_4_@mPEG-Ag hybrid-NPs demonstrate outstanding antibacterial efficacy against both types of (Gram-negative and Gram-positive) pathogens, primarily due to their synergistic interaction with bacterial cell walls. This cell contact interaction disrupts the bacterial outer membrane by inducing elevated ROS levels under visible light irradiation, combined with membrane penetration and controlled release of Ag^+^ ions. These multiple mechanisms collectively confer a versatile therapeutic profile, which is conducive to efficient bacterial elimination. Computational (in silico) analyses further corroborate the experimental (in vitro) outcomes, thereby strongly validating the observed antibacterial activities. Notably, the hybrid-NPs exhibit extremely low cytotoxicity, which strongly supports their harmless and effective biomedical use. Therefore, these multi-functional antibacterial hybrid-NPs hold robust potential for scalable production via a facile synthesis route using readily accessible materials, offering an effective solution for eradicating diverse drug-resistant microbes while reducing reliance on conventional antibiotics.

## Conclusion

In this study, mPEG- and silver-functionalized magnetite NPs (Fe_3_O_4_@mPEG-Ag NPs) have been developed via a serial coprecipitation synthesis technique to realize an excellent synthetic bactericide. Optical absorption spectra and the proposed charge transport mechanism indicate that an interfacial electric field between Fe_3_O_4_ and Ag across the conductive mPEG layer improves the transport efficiency of PCCs, thereby enhancing the formation of ROS in the system. Free radical scavenging experiments and ESR analyses ensured elevated levels of ^•^O_2_^−^, ^•^OH, and h^+^ active species under visible light illumination. The in vitro analysis revealed that Fe_3_O_4_@mPEG-Ag NPs demonstrated remarkable antibacterial efficacy, achieving a MIC of only 50 μg·ml^−1^ compared to that of pristine Fe_3_O_4_ (150 μg·ml^−1^), with performance comparable to the standard antibiotic CIP. The enhanced biocidal activity stems from the synergistic contact-based interaction of the hybrid-NPs, enabling effective bacterial surface anchoring, outer membrane disruption through elevated ROS generation, and intensified intracellular damage via mechanical piercing and controlled Ag^+^ ion release. Moreover, the hybrid-NPs demonstrate excellent biocompatibility, maintaining cell viability above 80% at concentrations up to 150 μg·ml^−1^, highlighting their potential for safe therapeutic applications. Finally, the computational molecular docking (in silico) analysis showed a strong correlation with the antibacterial results, confirming that the synthesized NPs exhibit potent inhibitory potential against key bacterial enzymes. These findings highlight the potential of Fe_3_O_4_@mPEG-Ag hybrid NPs in advancing the frontier of innovative drugs as viable alternatives to conventional antibiotics, thereby mitigating the high risks associated with antibiotic misuse.

## Materials and Methods

### Reagents and pathogenic cultures

Ferrous chloride tetrahydrate (FeCl_2_·4H_2_O, purity ≥99.0 wt %), ferric chloride hexahydrate (FeCl_3_·6H_2_O, purity ≥99.0 wt %), carboxyl-terminated methoxy polyethylene glycol (mPEG-COOH, purity ≥95.0 wt %), ammonium hydroxide solution (NH_4_OH, purity 28% to 30%), silver nitrate (AgNO_3_, purity ≥99.5 wt %), and 95% ethanol (EtOH) were purchased from Sigma-Aldrich and used as received. The in vitro case–control study utilized 4 different drug-resistant bacterial strains, including *E. coli* (ATCC 25922), *K. pneumoniae* (ATCC 13883), *S. aureus* (ATCC 25923), and *E. faecalis* (ATCC 19433), which were obtained from the School of Biomedical Engineering, Guangzhou Medical University, China. Reagents and solvents used in the antibacterial and minimum inhibitory concentration (MIC) evaluations, including nutrient broth, nutrient agar, and phosphate-buffered saline (PBS), were sourced from Sigma-Aldrich. All reagents and solvents were of analytical grade and utilized as acquired, without undergoing any extra refinement step.

### Fabrication of Fe_3_O_4_@mPEG-Ag hybrid-NPs

**Fig. 11.**
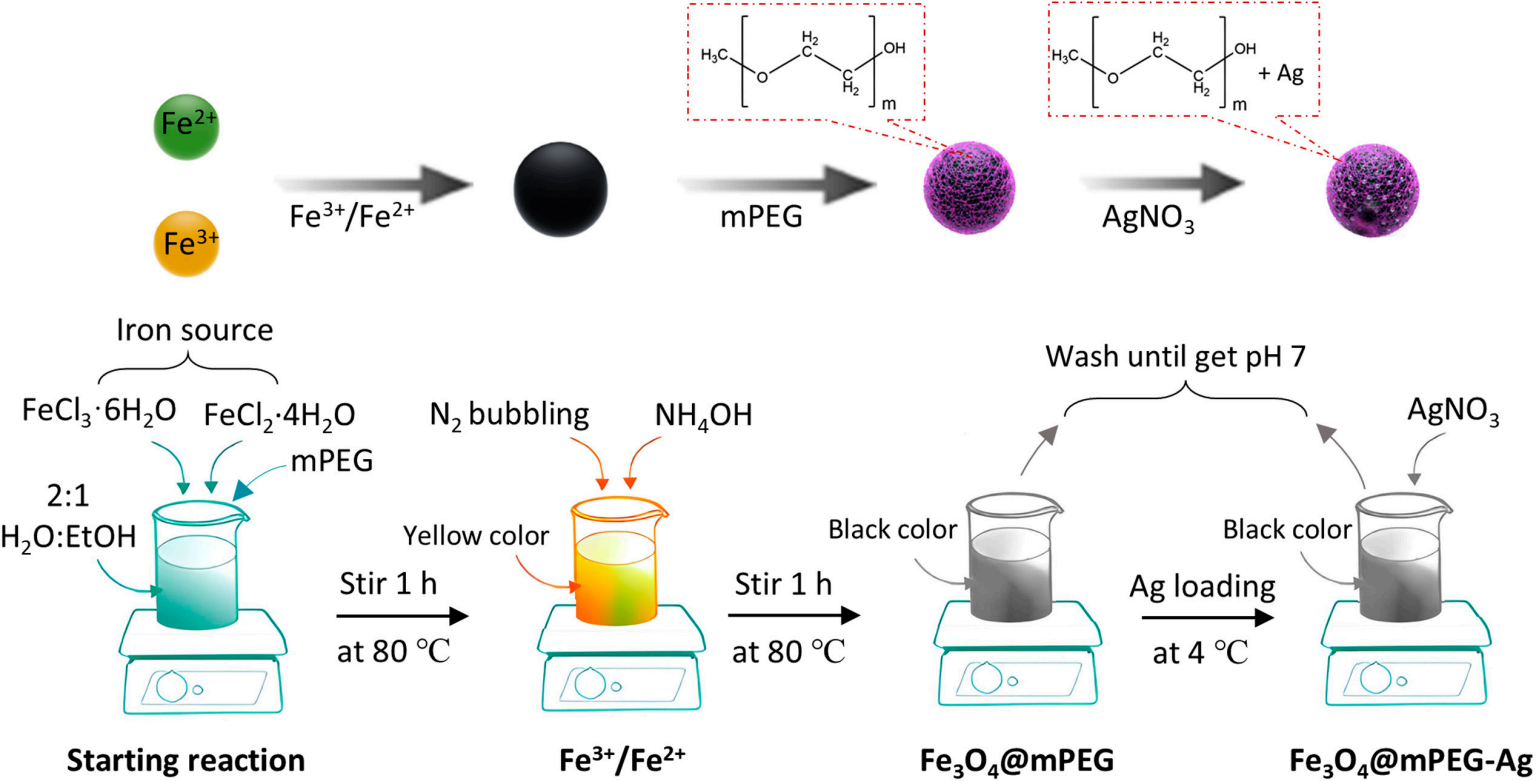
Schematic representation for the growth of Fe_3_O_4_@mPEG-Ag hybrid-NPs.

Fe_3_O_4_@mPEG-Ag NPs were successfully developed using the following synthesis procedure, as outlined in the schematic diagram of Fig. 11. In a typical synthesis method, 100 mg of mPEG, 200 mg (0.740 mmol) of FeCl_3_·6H_2_O, and 100 mg (0.503 mmol) of FeCl_2_·4H_2_O (molar ratio 1:2:1) were respectively dissolved in 100 ml of a 2:1 v/v mixture of deionized (DI) water and 95% EtOH. The solution temperature was gradually raised from room temperature (RT) (25 ± 1 °C) to 80 °C and then stirred under a gentle nitrogen (N_2_) bubbling (∼20 cm^3^·min^−1^) atmosphere for 20 min. This inert environment prevents oxidation of Fe^2+^ ions, thereby maintaining the desired Fe^2+^/Fe^3+^ ratio for Fe_3_O_4_ formation, and the gentle bubbling avoids turbulence, which can otherwise induce NP agglomeration. The Fe^2+^/Fe^3+^ redox chemistry enables the initial nucleation of Fe_3_O_4_ and later contributes to the in situ reduction of Ag^+^ ions during hybrid-NP formation. Next, 5 to 8 ml of NH_4_OH solution were added at a rate of 5 ml·min^−1^ to initiate the formation of Fe_3_O_4_ NPs (adjusting the pH value ~9 to 10) followed by continuous stirring for an additional hour to complete the reaction. The black precipitates were magnetically separated, and the supernatant was discarded and washed at least 5 times. Each washing cycle utilized 300 ml of double-distilled water, followed by magnetic separation to remove unreacted ions and residual ammonia. The precipitates were then rinsed with 300 ml of 95% EtOH to eliminate organic residues and promote dehydration. Washing was continued until the supernatant reached a neutral pH (~7), ensuring complete removal of alkaline and ionic contaminants. The resulting Fe_3_O_4_@mPEG NPs were resuspended in 20 ml of DI water for subsequent hybridization. To synthesize Fe_3_O_4_@mPEG-Ag hybrid-NPs, a 300-ml transparent [Ag(NH_3_)_2_]^+^ ion aqueous solution was formulated by diluting 600 mg of AgNO_3_ in a 2:1 mixture of DI water and 95% EtOH, followed by the addition of 2 ml of NH_4_OH to the AgNO_3_ solution. Subsequently, 15 ml of Fe_3_O_4_@mPEG suspension was injected into above 300 ml of [Ag(NH_3_)_2_]^+^ ion aqueous solution and kept magnetically stirred in an ice bath at a speed of 800 rpm for 1 h. During this process, Fe^2+^ ions in Fe_3_O_4_ serve as mild reducing agents, transferring electrons to Ag^+^ ions to nucleate and grow metallic Ag NPs on the Fe_3_O_4_@mPEG surface. Fe^2+^ is partially oxidized to Fe^3+^, while Fe^3+^ maintains the structural integrity of the Fe_3_O_4_ core. Finally, Fe_3_O_4_@mPEG-Ag NPs were collected by centrifugation (7,000 rpm, 5 min), thoroughly rinsed 3 times with DI water, and subsequently air-dried at 65 °C for 14 h. Importantly, the final product yield was measured after the drying process, which consistently reached approximately 0.410 g, indicating the efficiency and reproducibility of the synthesis process. For comparison, pristine Fe_3_O_4_ and Fe_3_O_4_@mPEG were synthesized following the same procedure, differing only in the exclusion of mPEG and Ag reagents in the synthesis of respective Fe_3_O_4_ and Fe_3_O_4_@mPEG counterparts.

### Characterization

An x-ray diffraction (XRD) study was conducted to identify the phase structure of the materials using a JEOL, SmartLab SE diffractometer, scanning over a 2θ range of 10° to 80° utilizing Cu-Kα x-rays (λ = 0.1540 nm). Functional group investigation was conducted through a Fourier transform infrared spectrometer (FTIR; Nicolet Protégé 460), operated in the transmission mode from 400 to 4,000 cm^−1^, using KBr discs. Morphological investigation and elemental mapping were conducted using transmission electron microscopy (TEM; JEOL JEM-2100 Multi-purpose Electron Microscope) and a high-angle annular dark field scanning transmission electron microscopy (HAADF-STEM), respectively. Elemental composition was analyzed via energy-dispersive spectroscopy (EDS) integrated with a Thermo Scientific Z microscope, optimized in nanoprobe mode at 190 kV, and equipped with a camera length of about 380 mm. Selected-area electron diffraction (SAED) patterns were collected to determine the crystallinity of the NPs. For sample preparation, each diluted suspension (1:2, suspension:EtOH) was placed onto a holey carbon-coated copper grid. The size distribution and the nature of the surface charge (zeta potential) of the hybrid-NPs were characterized through dynamic light scattering (DLS) with a Brookhaven ZetaPALS analyzer to evaluate their particle size, colloidal stability, and dispersion properties. Optical absorption characteristics were obtained using a PerkinElmer Lambda 20 ultraviolet (UV)–visible–NIR spectrometer, operated in a spectral range of 185 to 3,400 nm. Photoluminescence (PL) spectra were recorded using an F-7100 fluorescence spectrophotometer (Hitachi, Japan), covering a broad excitation range (*E*_x_: ~200 to 900 nm) and emission range (*E*_m_: ~220 to 900 nm), operated at a photomultiplier tube (PMT) voltage setting of 400 V. The photoelectrochemical measurements were conducted using a CHI660E electrochemical workstation, equipped with custom-fabricated quartz cells containing 3 electrodes. A Horiba FluoroMax-4 spectrofluorometer, utilizing a Xenon flash lamp, was employed to acquire fluorescence spectra. The concentration of Ag^+^ leaching was measured through an inductively coupled plasma–optical emission spectroscopy (ICP-OES) setup (iCAP 7000 Plus Series, Thermo Scientific). ROS detection was carried out via an electron spin resonance spectrometer (ESR), using a Magnettech ESR5000 system (Bruker, Germany), with all measurements conducted at RT (RT ≈ 24 ± 1 °C). Protein levels and catalase (CAT) activities were assessed using enzyme-linked immunosorbent assay (ELISA) accomplished on a Thermo Fisher Varioskan LUX multi-mode plate reader.

### Photoactivation protocol

All experiments under visible light irradiations were conducted following a modified procedure based on a previously reported procedure [33]. Photoactivation was performed directly on agar plates containing 6-mm wells loaded with antibacterial NP suspensions. The plates were positioned 10 cm below a 300-W high-intensity Xenon arc tuber (model: 66483-300XF-R22) emitting ozone-free visible light with an intensity of 1,000 W·m^−2^. The lamp assembly (Newport #67001) contained an F/2.2 fused silica condenser and a rear reflector, with a correction factor of 1.6 applied for intensity adjustment. To ensure uniform irradiation and minimize spatial intensity gradients, a collimated light beam (∼33 nm in diameter) was aligned to cover the inoculated area of each well. A recirculating water filter maintained at a constant temperature was employed to remove infrared (IR) radiation and prevent overheating during exposure. Additionally, UV light was selectively filtered using a 400-nm long-pass cutoff filter (Oriel #FSQ-GG400), which effectively blocked lower wavelengths. The radiation intensity was calculated based on an average source irradiance of 30 mW·m^−2^·nm^−1^, measured at a 0.5-m working distance. The calculation accounted for the relationship between radiant power and exposure time to determine the total radiant energy delivered. Only the incident radiation falling within the spectral window allowed by the ∼400-nm UV cutoff filter and corresponding to the semiconductor’s optical absorption band was utilized during the light exposure. All experiments involving visible light activation were performed at a fixed 10-cm distance from the light source, with the irradiation intensity determined as 1.6 J·cm^−2^·s^−1^ using a Laser Mate power meter (Coherent).

### Evaluation of antibacterial properties

#### Preparation of bacterial suspension

Four bacterial strains, such as *E. coli* (ATCC 25922), *K. pneumoniae* (ATCC 13883), *S. aureus* (ATCC 25923), and *E. faecalis* (ATCC 19433), were selected as representative bacterial strains for antibacterial evaluation. Each strain was initially cultured in 5 ml of sterilized Luria–Bertani (LB) broth and incubated overnight at 37 °C in a shaking incubator at 170 rpm. To prepare the working bacterial suspensions, 2 ml of each culture was centrifuged at 3,500 rpm for 5 min at 20 °C to collect the bacterial cell pellet. The pellets were repeatedly washed with PBS (containing 75% NaCl) to remove residual culture medium. The final bacterial suspensions were then diluted to the required concentrations according to the specific experimental requirements.

#### Antibacterial susceptibility test using the agar well diffusion method

The agar well diffusion method was employed to evaluate the antibacterial efficiency of the developed materials following a previously described protocol with minor modifications [68]. Typically, each bacterial suspension was standardized to 9 × 10^6^ colony-forming units (CFU)·ml^−1^ according to 0.5 McFarland turbidity standard and subsequently diluted in PBS to reach a final concentration of nearly 9 × 10^7^ CFU·ml^−1^, based on optical density (OD_600_) measurements. Subsequently, 75 μl of each bacterial suspension was aseptically inoculated onto Mueller–Hinton agar (MHA) plates, uniformly spread using sterile glass beads, and allowed to dry completely. Wells of 6 mm in diameter were then bored into the agar surface, and 75 μl of NP suspensions in PBS was added at different concentrations: 150 μg·ml^−1^ (Fe_3_O_4_), 100 μg·ml^−1^ (Fe_3_O_4_@mPEG), 50 μg·ml^−1^ (Fe_3_O_4_@mPEG-Ag), 25 μg·ml^−1^ (Fe_3_O_4_@mPEG-Ag), 12.5 μg·ml^−1^ (Fe_3_O_4_@mPEG-Ag), and 0 μg·ml^−1^ (Fe_3_O_4_@mPEG-Ag). After loading the wells, the plates were left undisturbed for 15 min to allow diffusion of the NP suspensions into the agar medium. Each well was then subjected to visible light irradiation (λ > 400 nm) using a 300-W Xenon arc lamp positioned 10 cm above the agar surface for 15 min. The irradiation was performed under controlled temperature conditions, while parallel plates were maintained in the dark (-Ve controls) to assess light-dependent effects. This approach allows direct assessment of light-triggered ROS-mediated antibacterial mechanisms. Post-irradiation, the standard antibiotic CIP (25 μg·ml^−1^) was loaded into separate wells on the same plates to prevent its intrinsic antibiotic activity. All plates were subsequently incubated at 37 °C for 24 h, after which zones of inhibition (ZOIs) were measured in millimeters to quantify antibacterial efficacy. For quantitative assessment, post-incubation absorbance readings were taken using a Gen 5 Bio-Tek microplate photometer integrated with a shaker and thermostat, enabling accurate measurement of bacterial growth inhibition in each well. Noticeable pathogenic growth inhibition was observed for all NP-treated samples, excluding the untreated control group (0 μg·ml^−1^). The CIP served as the positive control, demonstrating pronounced bacterial growth inhibition zones and validating the assay reliability. All experimental results were analyzed following Clinical and Laboratory Standard Institute (CLSI) guidelines to ensure methodological accuracy and clinical relevance for their potential application.

### MIC study

To determine the MICs of the antibacterial samples, a micro-broth dilution assay was conducted in 96-well microtiter plates, following an established procedure [41]. The employed standard MIC protocol began with a 2 mg·ml^−1^ stock solution subjected to serial dilution, yielding final concentrations between 2 and 0.0643 mg·ml^−1^. Each pathogenic inoculum was prepared to match turbidity to the 2.0 McFarland standard, corresponding to approximately 9 × 10^6^ to 9 × 10^7^ CFU·ml^−1^. In the microtiter plate, 0.25 ml of LB broth comprising both pathogenic strains and different concentrations of the antibacterial compounds (0, 12.5, 25, 50, 100, and 150 μg·ml^−1^) was filled to the top rows. Next, 0.25 ml of LB broth was supplemented with CIP at 25 μg·ml^−1^ dose and each pathogenic strain was added into the subsequent 4 rows. For comparison, blank LB suspension (without antibacterial compounds or bacterial culture) was added into the consequent row. In the same manner, LB broth encompassing either only antibacterial agents or pathogenic strains was added to subsequent rows. The microplates were incubated aerobically at 37 °C for 20 to 24 h to facilitate optimal bacterial growth and assess the antibacterial activity. Experimental setups included distinct groups: bacterial suspension without any bactericidal agents (blank group), CIP-treated suspensions (positive control), and antibacterial agent-treated samples but without pathogens (negative control). The MIC was well-defined as the lowest amount of bactericidal substance required to visibly hinder the progression of pathogenic organisms.

### ROS analysis

To assess the formation of ROS radicals in the well-developed hybrid-NPs, a series of selective molecular probe were employed, including DCFH-DA (2′,7′-dichlorohydihydrofluorescein diacetate) for general ROS detection, 3-CCA (comarine-3-carboxylic acid) for hydroxyl (OH^•^) radicals’ identification, and p-NBT (p-nitroblue tetrazolium chloride) for superoxide (^•^O₂^−^) anion quantification. Fluorescence detection was conducted using respective excitation (*E*_x_: 480 nm) and emission (*E*_m_: 525 nm) wavelengths, specifically for identifying OH^•^ species. The generation of OH^•^ radicals in the samples was assessed following treatment with 2 mM 3-CCA under both visible light illumination and dark conditions, using Fe_3_O_4_@mPEG-Ag NPs. ROS detection was also performed with a 3-CCA sensor containing Fe_3_O_4_@mPEG-Ag NPs under visible light. Additionally, the time-resolved generation of ROS was evaluated by monitoring the reduction of p-NBT to formazan, indicating the presence of ^•^O_2_^−^ anions. To quantify the total ROS level in all 3 sample types, DCFH-DA was used as a fluorescent probe. The emission spectra of the well-treated reactions were recorded by employing an EDINBURG fluorescence spectrophotometer (FS5) to quantitatively evaluate the fluorescence intensities.

### In vitro hemolysis and cytotoxicity evaluation

The as-synthesized Fe_3_O_4_@mPEG-Ag hybrid-NPs were subjected to comprehensive in vitro assessments to evaluate their hemolytic activity and cytotoxicity effects, using respective rat red blood cells (RBCs) and mouse embryonic fibroblast L929 cells as representative cell models. The rat RBCs were purchased from Beijing Vital River Laboratory Animal Technology Co. Ltd. (Beijing, China), while the mouse embryonic fibroblast L929 cells were obtained from volunteers of the school of Biomedical Engineering (Guangzhou Medical University). All procedures involving the use of biological materials were conducted in accordance with the ethical guidelines and approved protocols established by the Ethics Committee of Guangzhou Medical University, China. For hemolytic activity evaluation, fresh rat blood was first centrifuged with 2% ascorbic acid for 10 min and washed 3 times with PBS. Subsequently, 1 ml of the diluted erythrocyte suspension was aliquoted into 5 separate sample tubes for further testing. Subsequently, 100 μl of PBS, varying concentrations of the hybrid-NPs (50, 100, and 150 μg), and 0.1% Triton X-100 (0.1 ml) were inserted into the prepared tubes, where the first and fifth tube was designated as the negative (-Ve) and positive (+Ve) control groups of the experiment, respectively. After incubation at 37 °C for 5 h, the erythrocyte suspensions were centrifuged at 3,000 rpm for 5 min, and the absorbance of the collected supernatant was recorded at 545 nm employing a UV–vis–NIR spectrometer.

The hemolysis ratio (%) for all treated samples was calculated using the following equation.

$$\text{Hemolysis ratio}\;\left(\%\right)=\frac{A_{S}-A_{P}}{A_{T}-A_{P}}\times 100\%$$ (13)

Here, *A*_S_, *A*_P_, and *A*_T_ represent the supernatant fraction absorbance of hybrid-NP sample, PBS, and Triton, respectively.

In addition, the in vitro cytotoxicity of the synthesized hybrid-NPs was evaluated against mouse embryonic fibroblast L929 cells at varying concentrations (0 to 150 μg·ml^−1^) using the 3-(4,5-dimethylthiazol-2-yl)-2,5-diphenyltetrazolium bromide (MTT) assay and fluorescence staining, as described in a previously reported protocol. The percentage of the viable cells was computed based on the given equation.

$$\text{Cell viability}\;\left(\%\right)=\frac{A_{S}-A_{C}}{A_{C}}\times 100\%$$ (14)

Here, *A*_C_ and *A*_S_ denote the optical absorbance of the control group (untreated) and the different concentrations of the treated hybrid-NP sample, respectively.

### Computational molecular docking analysis

A computational analysis involving molecular docking (in silico) was performed on the synthesized samples, targeting 2 key bacterial enzymes: *β-lactamase* from *E. coli* [Protein Data Bank (PDB): 4KZ9, 1.72 Å] and *DNA gyrase* from *S. aureus* (PDB: 5MMN, 1.90 Å) bacterial strains. These enzymes play essential roles in bacterial functions, such as cell wall construction and DNA replication, making them essential therapeutic candidates for therapeutic intervention. The 3-dimensional (3D) structures of the enzymes were retrieved from the PDB and processed using the Molecular Operating Environment (MOE) software suite. Prior to docking, both proteins and ligand structures were optimized through 3D protonation and energy minimization, applying the MMFF94X force field with a 0.05 gradient. The docking procedure was carried out using the standard configuration provided by MOEs, which included the Triangle Matcher algorithm for legend placement, Forcefield for structural refinement, London dG for the initial scoring phase, and GBVI/WSA for the second round of scoring. A total of 10 different ligand conformations were produced, and the top-ranking ones that were determined by docking scores were chosen for evaluating protein–ligand binding interaction analysis. The resulting molecular interactions were then visualized using the Pymol software program [33,50].

### Statistical analysis

To ensure the accuracy of the results, all experimental data were expressed as the means ± SD, with *n* = 3 for each experimental group, reflecting a consistent number of biological replicates. Statistical significance was determined using *P* values (denoted as *P*), where *P* < 0.05 (*P) indicated significance, *P* < 0.01 (**P) denoted strong significance, and *P* < 0.001 (***P) reflected high statistical confidence. Comparison between groups was conducted using a *t* test in Origin 2021 software.

## Data Availability

The datasets generated and/or analyzed during the current study are available from the corresponding authors upon request.

## Supplementary Materials

Supplementary 1 Supplementary Text Figs. S1 to S7 Tables S1 and S2 References
