# Erratum to “Synergistic Antibacterial Activity of Fe_3_O_4_@mPEG-Ag Nanoparticles with Molecular Docking Analyses”

**DOI:** 10.34133/bmef.0240

**Published:** 2026-04-21

**Authors:** Basit Ali Shah, Hongguo Zhu, Asma Sardar, Yuan Gu, Syed Taj Ud Din, Kashif Naseem, Xinyan Wu, Bin Yuan, Bin Yang

**Affiliations:** ^1^ School of Biomedical Engineering, The Fourth Affiliated Hospital of Guangzhou Medical University, Guangzhou Medical University, Guangzhou 511436, China.; ^2^ School of Materials Science and Engineering, South China University of Technology, Guangzhou 510641, China.; ^3^ Department of Chemistry, Fatima Jinnah Women University, Rawalpindi 46000, Pakistan.; ^4^ Department of Physics, Dongguk University, Seoul 03760, Korea.; ^5^ School of Material and Environmental Engineering, Hunan University of Humanities Science and Technology, Loudi 417000, China.; ^6^ College of Food Science and Nutritional Engineering, China Agricultural University, Beijing 100083, China.

Since its initial publication, the layout of this article has been modified [1]. This involves reordering the manuscript sections and subsequent renumbering of the reference list to maintain sequential order. These updates are strictly organizational; the scientific findings, data, and discussion remain identical to the original version.
