# Supplementary material for: Synergistic Antibacterial Activity of Fe3O4@mPEG-Ag Nanoparticles with Molecular Docking Analyses
Source: BME Front. 2025 Dec 26;6:0214. doi: 10.34133/bmef.0214 (PMC12741258; doi:10.34133/bmef.0214)
Supplement: Supplementary 1 — Supplementary Text Figs. S1 to S7 Tables S1 and S2 References [file bmef.0214.f1.docx]

**Supporting Information**

**Synergistic antibacterial activity of Fe_3_O_4_@mPEG-Ag nanoparticles** **with molecular docking analyses**

**Basit Ali Shah^1,2,^**^†^**, Hongguo Zhu^1,^**^†^**, Asma Sardar^3^, Yuan Gu^1^, Syed Taj Ud Din^4^, Kashif Naseem^5^, Xinyan Wu^6^, Bin Yuan^2 ⃰^, Bin Yang^1^ ⃰**

^1^School of Biomedical Engineering, The Fourth Affiliated Hospital of Guangzhou Medical University, Guangzhou Medical University, Guangzhou 511436, China

^2^School of Materials Science & Engineering, South China University of Technology, Guangzhou 510641, China

^3^Department of Chemistry, Fatima Jinnah Women University, Rawalpindi 46000, Pakistan

^4^Department of Physics, Dongguk University, Seoul 03760, Korea

^5^School of Material & Environmental Engineering, Hunan University of Humanities Science and Technology, Loudi 417000, China

^6^College of Food Science and Nutritional Engineering, China Agricultural University, Beijing 100083, China

†These authors contribute equally to this article.

**⃰Corresponding authors:** [bin.yang@gzhmu.edu.cn](mailto:bin.yang@gzhmu.edu.cn); apsheng@scut.edu.cn

## **Quantification of Ag^+^ release**

To assess the Ag^+^ release behavior, 50 mg of Fe_3_O_4_@mPEG-Ag NPs were sealed in a dialysis membrane (molecular weight cut-off ~3500 kDa) and immersed in 800 mL of distilled water within a 1000 mL beaker. The dialysis membrane containing nanoparticles was maintained under continuous magnetic stirring at room temperature (24 ± 1 ℃), with the light-exposed subject to uninterrupted UV-visible irradiation for 24 h to stimulate the light-triggered Ag^+^ release. In contrast, the control sample was completely wrapped in aluminum foil to prevent any light exposure and served as the dark reference. At predetermined intervals of each 2-hour, 5 mL samples of surrounding water were collected and stored for further analysis. The concentration of released Ag^+^ was quantified using an ICP-OES setup (iCAP^TM^ 7000 Plus Series, Thermo Scientific).

**Fluorescence staining of live/dead bacterial cells**

To assess bacterial cell viability, a fluorescence-based live/dead staining assay was performed, using bacterial suspensions prepared with varying concentrations (0‒150 μg‧mL⁻¹) of NPs. Two different bacterial strains, such as Gram-negative‒*E. coli* and Gram-positive‒*S. aureus* were employed and each bacterial strain was adjusted to a dilution factor of 5 × 10^7^ CFU‧mL⁻¹. The samples were incubated with NPs under specified conditions and stained with a dual fluorescent dye system to distinguish live (green-fluorescent) and dead (red-fluorescent) cells, while the bacterial suspension without NPs (PBS) served as the control group. Following given treatment, bacteria were isolated via centrifugation at 3000 rpm for 10 minutes. The bacterial pellets were then double-stained using SYTO 9 and PI (propidium iodide) at a 5:5 μL ratio, with an incubation time of 40 minutes. After a 30-minute staining period, the samples were subsequently washed twice with PBS to eliminate any residual dye. Approximately 40 μL of each stained bacterial suspension was transferred to a confocal dish for analysis. The live and dead bacterial cells were observed and imaged using an inverted fluorescence microscope (Model: IFM, Olympus, 1X73), providing detailed insights into the effectiveness of the NPs treatments.

## **Morphological investigation of bacterial cells**

The surface morphology of bacterial strains treated with PBS, Fe_3_O_4_, Fe_3_O_4_@mPEG, and Fe_3_O_4_@mPEG-Ag NPs was examined using field emission scanning electron microscopy to evaluate the antibacterial impact of the synthesized NPs. The bacterial cells were separated by centrifuging from the prepared stock solution containing the bacterial strain and antibacterial NPs, followed by repeated rinsing with diluted NaCl solution. The isolated bacterial cells were fixed in 3% aqueous glutaraldehyde solution at 4 °C for 12 h and subsequently dehydrated in a graded series of anhydrous ethanol solutions (25%, 50%, 75%, and 100%), each for 15 minutes. The dehydrated samples were stored at -80 °C and subjected to cryodesiccation before imaging with FESEM to evaluate bacterial cell lysis, using the untreated bacterial cells as the blank control group.


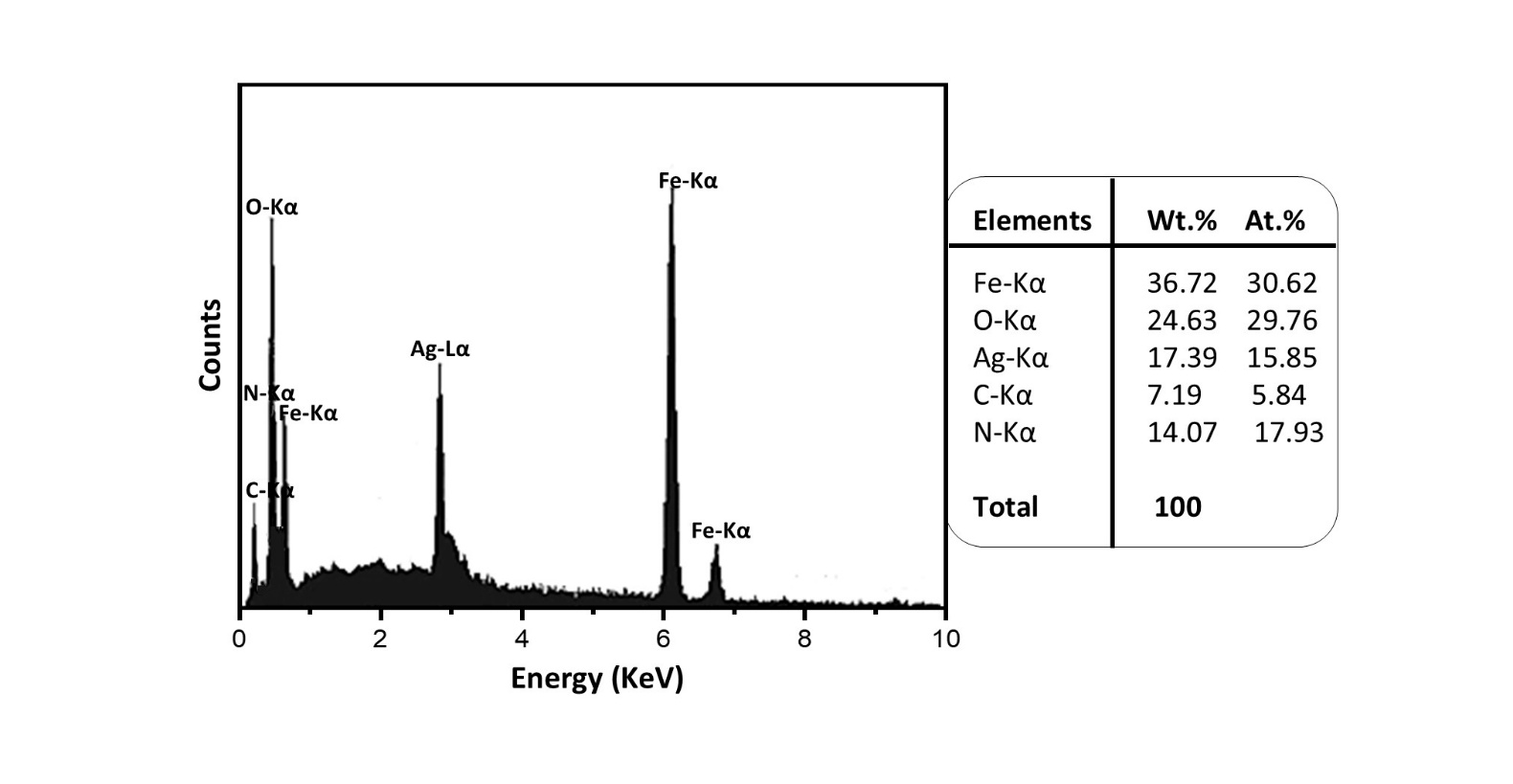


Figure S1 EDS profile associated by an inset table illustrating the elemental composition in percentage proportion of the expected elements in Fe_3_O_4_@mPEG-Ag hybrid-NPs.


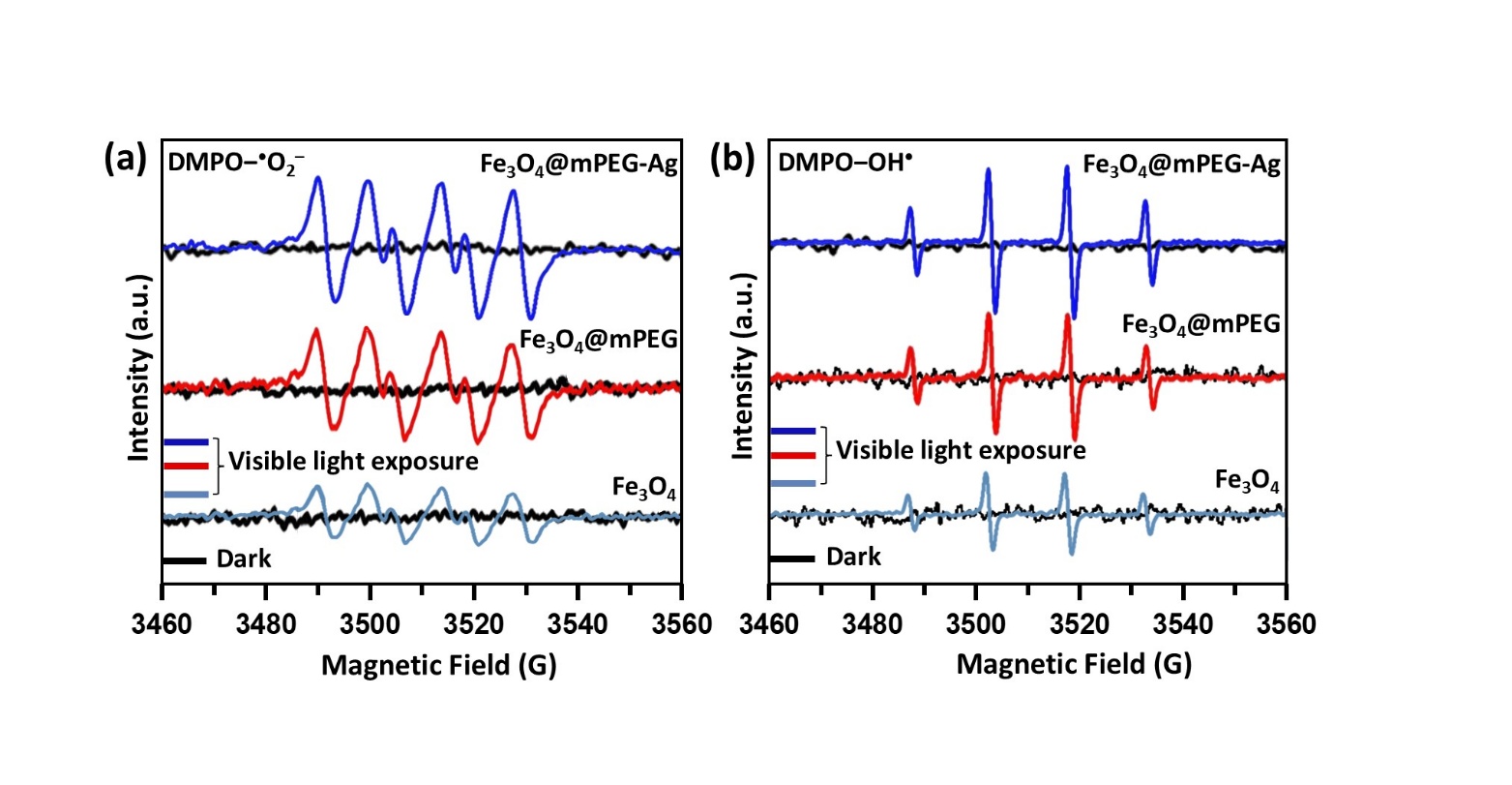


Figure S2 ESR spectra: (a) DMPO−^•^O_2_^–^, and (b) DMPO−OH^•^ for Fe_3_O_4_, Fe_3_O_4_@mPEG, and Fe_3_O_4_@mPEG-Ag hybrid-NPs samples under Dark conditions and 20 min of visible light irradiations


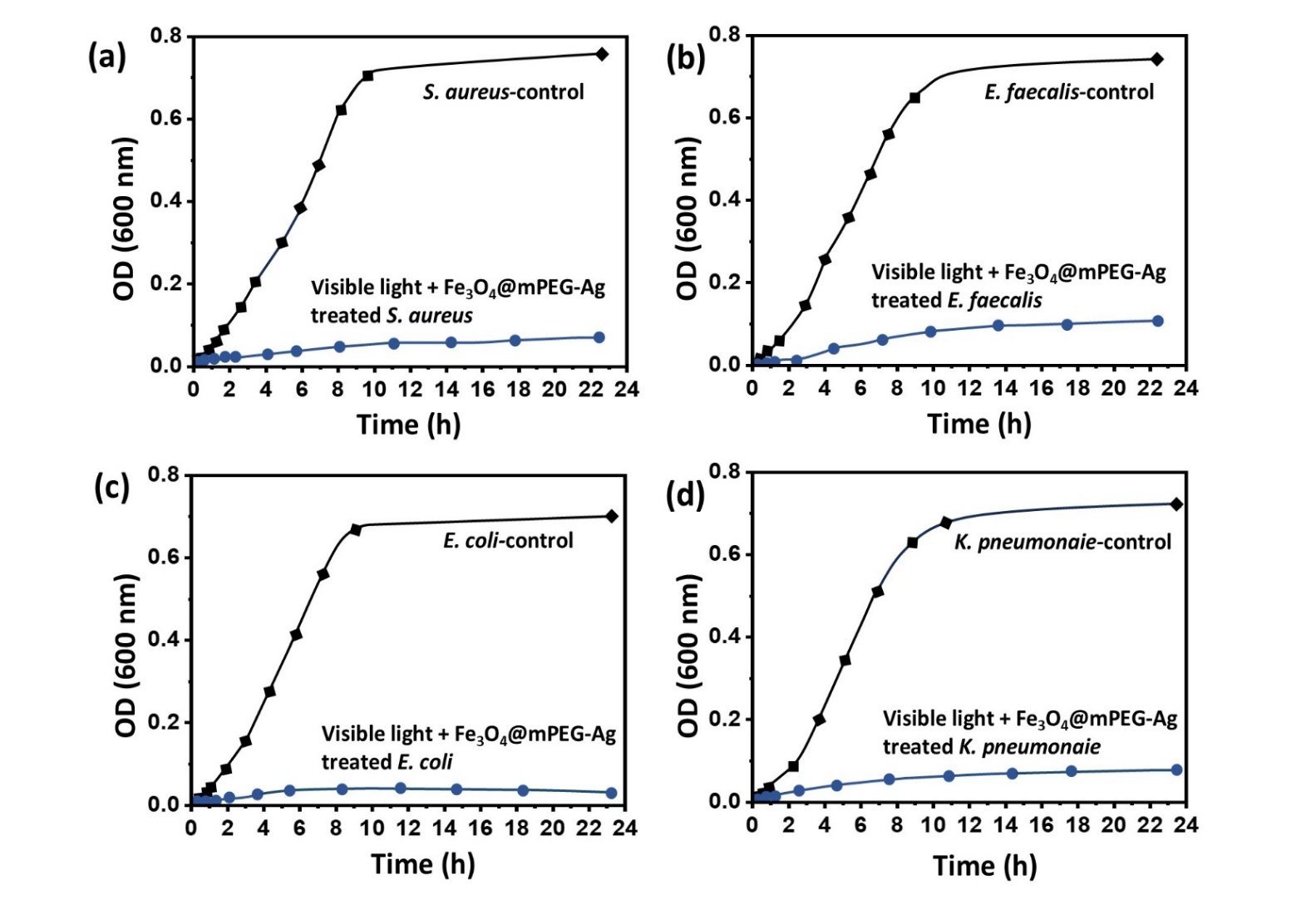


Figure S3 Corresponding growth profiles of four different bacterial strains: (a) *S. aureus*, (b) *E. faecalis,* (c) *E. coli, and* (d) K. pneumoniae with comparative analyses between untreated samples and those exposed to Fe_3_O_4_@mPEG-Ag hybrid-NPs under 1.6 J dose of visible-light irradiations

Figure S4a presents the antibacterial performance of the prepared samples, illustrating the zones of inhibition (ZOI) against all four tested pathogens under dark conditions at varying concentrations, and are further quantified in the bar graph of Figure S4b. The antibacterial efficacy was evaluated over 0‒150 μg‧mL^-1^ concentration. At the baseline concentration (0 μg‧mL^-1^, labeled 1), no single ZOI was observed, indicating that all pathogenic strains proliferated freely in the absence of NPs treatment. Upon exposure to antibacterial NPs, clear inhibition zones have emerged, demonstrating their effectiveness in suppressing bacterial proliferation. At higher concentrations, especially 75 µL of 150 μg‧mL^-1^ for pristine Fe_3_O_4_ NPs (labeled 2) and 75 µL of 100 μg‧mL^-1^ for Fe_3_O_4_@mPEG NPs (labeled 3), the tested strains (*K. pneumoniae, E. coli, E. faecalis, and S. aureus*) showed resistant and intermediate ZOI diameters of (9.20 ± 0.8 mm), (7.74 ± 1.0 mm), (8.53 ± 1.1 mm), and (6.9 ± 07 mm) for Fe_3_O_4_, and (11.59 ± 1.3 mm), (11.0 ± 0.9 mm), (11.45 ± 1.2 mm), and (9.98 ± 08 mm) for Fe_3_O_4_@mPEG, respectively. In contrast, the Fe_3_O_4_@mPEG-Ag NPs at a lower concentration of 75 μL of 50 μg‧mL^-1^ (labeled 4) exhibited substantially larger inhibition zone diameters of (15.42 ± 1.2 mm), (16.94 ± 0.9 mm), (17.42 ± 1.2 mm), and (15.61 ± 1.1 mm) than its counterparts. The +Ve control group‒ciprofloxacin (75 μL of 25 μg‧mL^-1^, labeled 5) displayed excellent antibacterial activity with respective zone inhibition values of 28.25 ± 1.1 mm (*K. pneumonia*)*,* 29.26 ± 0.9 mm (*E. coli*), 29.81 ± 1.0 mm (*E. faecalis*)*,* and 27.52 ± 0.8 mm (*S. aureus*). Furthermore, the concentration-dependent antibacterial performance of the resultant Fe_3_O_4_@mPEG-Ag NPs is evident in the reversed order from samples 6 to 4. Detailed results corresponding to each concentration and ZOI are summarized in Table S1. The comparative analysis confirmed that the ZOI values of Fe_3_O_4_@mPEG-Ag hybrid-NPs exceed the CLSI criteria even under dark conditions, further confirming their robust and broad-spectrum antibacterial performance.


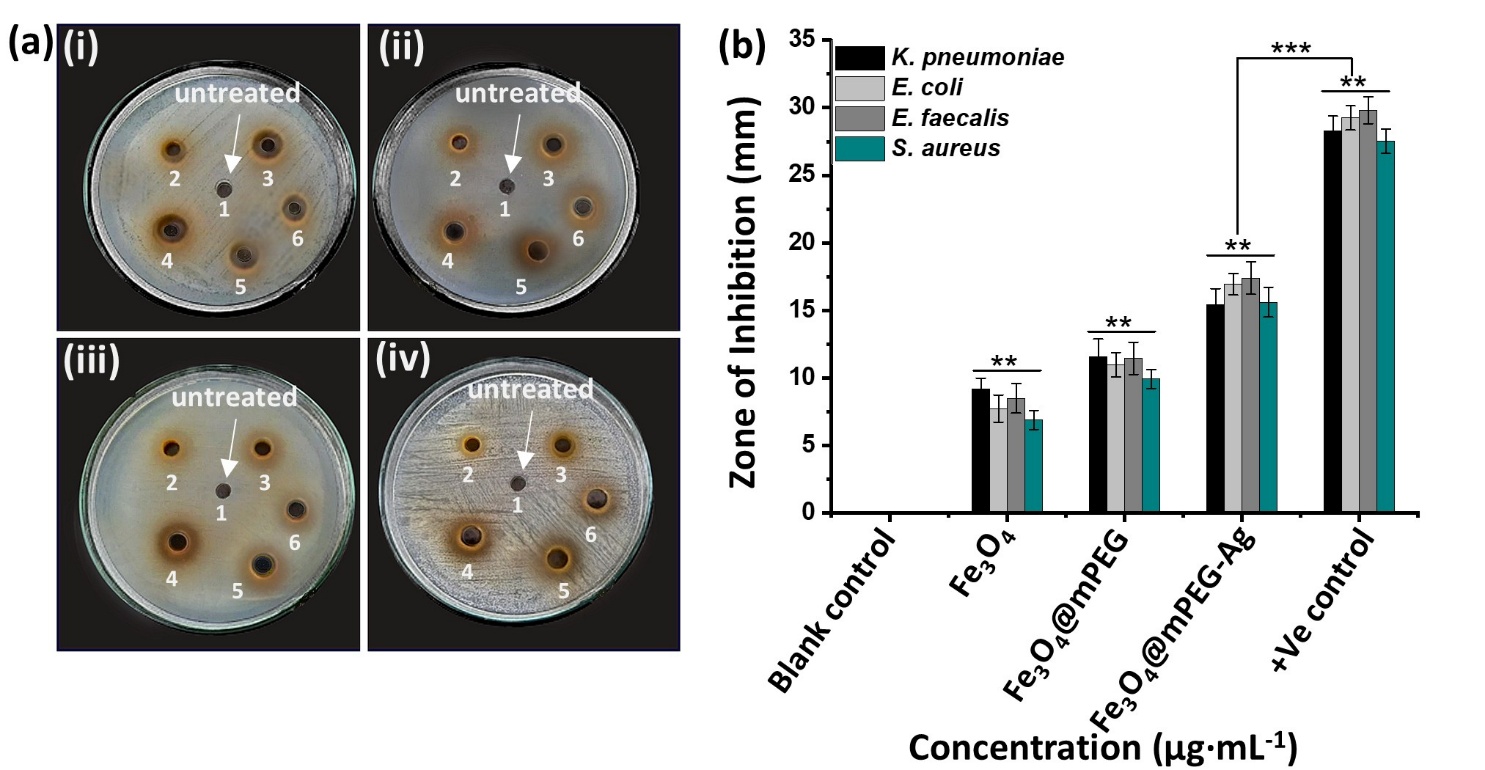


Figure S4 (a) Antibacterial performance of the developed samples at different concentrations targeting (i) *K. pneumoniae*, (ii) *E. coli,* (iii) *E. faecalis*, and (iv) *S. aureus* strains under dark conditions. The treatment groups included: 1. untreated samples (blank controls), 2. Fe_3_O_4_-treated samples at 150 μg‧mL^-1^, 3. Fe_3_O_4_@mPEG-treated samples at 100 μg‧mL^-1^, 4. Fe_3_O_4_@mPEG-Ag-treated samples at 50 μg‧mL^-1^, 5. Fe_3_O_4_@mPEG-Ag-treated samples at 25 μg‧mL^-1^, and 6. Fe_3_O_4_@mPEG-Ag-treated sample at 12.5 μg‧mL^-1^ (b) bar diagrams illustrating the corresponding ZOI diameter in millimeters for all pathogenic strains treated with various concentrations of antibacterial agents: 0 μg‧mL^-1^‒Fe_3_O_4_@mPEG-Ag NPs (blank control), 150 μg‧mL^-1^‒Fe_3_O_4_ NPs, 100 μg‧mL^-1^‒Fe_3_O_4_@mPEG NPs, 50 μg‧mL^-1^‒Fe_3_O_4_@mPEG-Ag NPs, and 25 μg‧mL^-1^‒ciprofloxacin-CIP (positive control). Initial comparisons were made against *K. pneumonia* and subsequently followed by intergroup comparisons among the 4 treatments at different concentrations. Statistical significance was represented as p*** < 0.001, p** < 0.01, and p* < 0.05.

Table S1 The MIC and mean diameters (mm) ZOI of the bank control and Fe_3_O_4_@mPEG-Ag NPs-treated samples against both Gram-negative and Gram-positive pathogenic strain

| **Concentration (µg mL^-1^)** | **ZOI**  **(mm) ± SD**  ***K. pneumoniae*** | **Sensitivity** | **Concentration (µg mL^-1^)** | **ZOI**  **(mm) ± SD**  ***E. coli*** | **Sensitivity** |
| --- | --- | --- | --- | --- | --- |
| **Gram-negative bacteria** | | | | | |
| Blank sample | 0.00 ± 0.00 | Nil | Blank sample | 0.00 ± 0.00 | Nil |
| Fe_3_O_4_@mPEG-Ag‒12.5 | 8.14 ± 1.0 | Resistant | Fe_3_O_4_@mPEG-Ag‒12.5 | 9.02 ± 1.2 | Resistant |
| Fe_3_O_4_@mPEG-Ag‒25 | 14.35 ± 0.8 | Intermediate | Fe_3_O_4_@mPEG-Ag‒25 | 14.00 ± 1.4 | Intermediate |
| Fe_3_O_4_@mPEG-Ag‒50 | 15.42 ± 1.2 | Intermediate | Fe_3_O_4_@mPEG-Ag‒50 | 16.96 ± 1.0 | Intermediate |
| **Concentration (µg mL^-1^)** | **ZOI**  **(mm) ± SD**  ***E. faecalis*** | **Sensitivity** | **Concentration (µg mL^-1^)** | **ZOI**  **(mm) ± SD**  ***S. aureus*** | **Sensitivity** |
| **Gram-positive bacteria** | | | | | |
| Blank sample | 0.00 ± 0.00 | Nil | Blank sample | 0.00 ± 0.00 | Nil |
| Fe_3_O_4_@mPEG-Ag‒12.5 | 7.53 ± 1.3 | Resistant | Fe_3_O_4_@mPEG-Ag‒12.5 | 8.43 ± 0.9 | Resistant |
| Fe_3_O_4_@mPEG-Ag‒25 | 14.35 ± 1.0 | Intermediate | Fe_3_O_4_@mPEG-Ag‒25 | 14.06 ± 1.3 | Intermediate |
| Fe_3_O_4_@mPEG-Ag‒50 | 17.77 ± 1.3 | Intermediate | Fe_3_O_4_@mPEG-Ag‒50 | 15.61 ± 1.1 | Intermediate |

Figure S5a illustrates the experimental setup used to evaluate the Ag^+^ release behavior from Fe_3_O_4_@mPEG-Ag NPs, where the hybrid-NPs were sealed in a dialysis membrane and immersed in distilled water. Figure 5b displays the time-dependent Ag^+^ release profiles over 24 h under continuous UV-visible light irradiation and in dark conditions. The light-exposed sample (blue curve) exhibits a markedly accelerated and higher Ag^+^ release compared to the dark sample (black curve), confirming the photoresponsive nature of the hybrid-NPs. Notably, the light-irradiated sample shows a sharp increase in the Ag^+^ concentration within the first 8 h, reaching approximately 145.6 ± 2.09 ppb, indicating a rapid release phase that is especially crucial for the early-stage eradication of biofilms, that are typically resistant to conventional therapies. In contrast, the dark condition-treated sample demonstrates a slower and declined Ag^+^ release profile, reaching only of 61.4 ± 1.072 ppb, which reflects passive diffusion in the absence of external stimulation. This dual-mode release behavior, comprising on an initial burst followed by sustained release provides a promising strategy for both immediate bacterial inactivation and prolonged infection control. Overall, the sustained Ag⁺ release under UV-visible light not only ensures enhanced biocidal performance but also maintains excellent biocompatibility, highlighting the therapeutic potential of Fe_3_O_4_@mPEG-Ag NPs for advanced anti-infective and anti-biofilm applications.


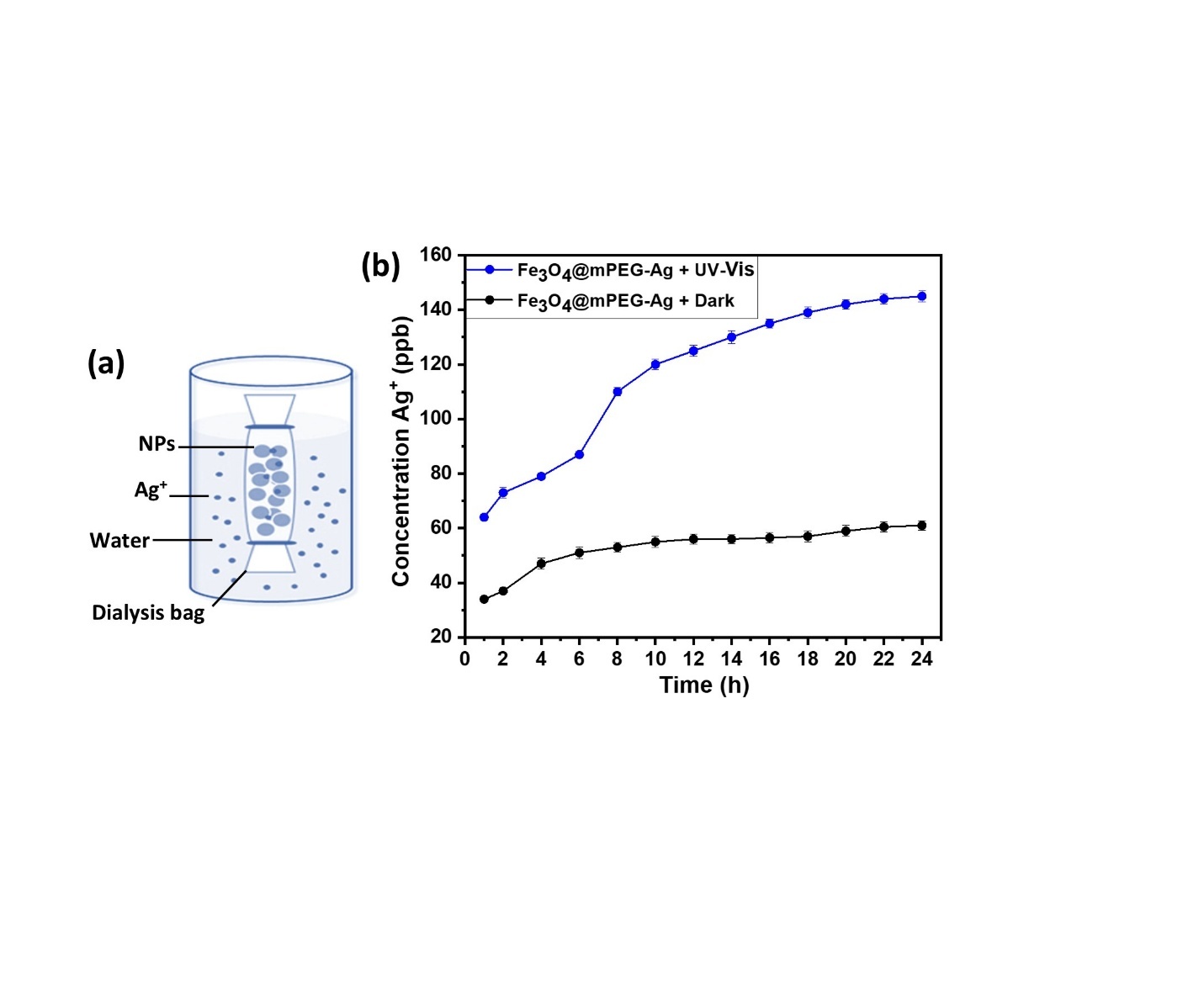


Figure S5 (a) Graphical illustration of the dialysis-based Ag^+^ ions leaching quantification, (b) Concentration of Ag^+^ release by Fe_3_O_4_@mPEG-Ag NPs through a dialysis membrane over different treatment in the presence (blue color) and absence (black) of UV-Visible light irradiation, respectively


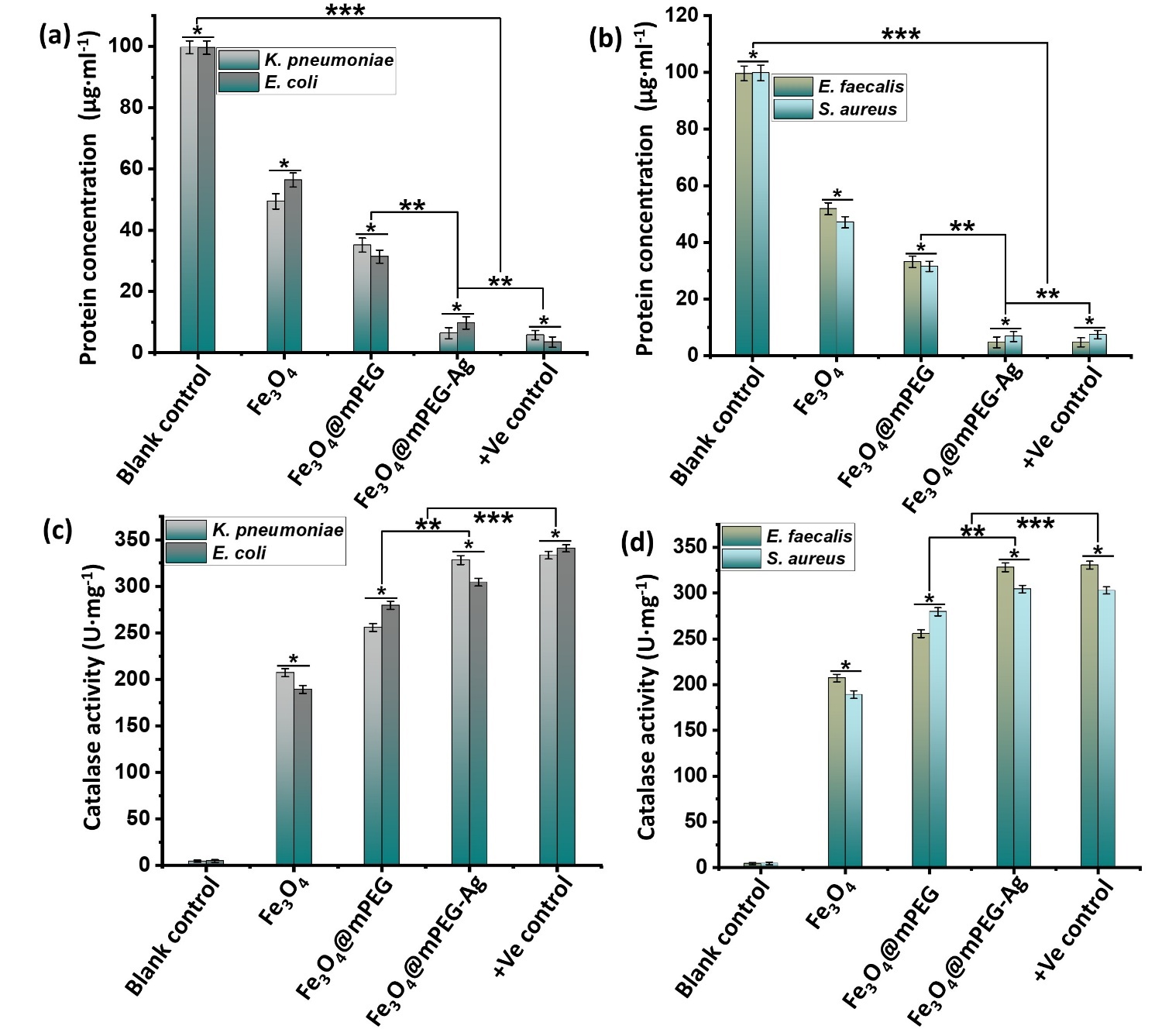


Figure S6 (a, b) Bar graphs of total protein levels and (c, d) catalase activity in response to treatment with varying concentrations (0‒150 μg‧mL^-1^) of antibacterial NPs targeting both Gram-positive and Gram-negative strains. Each dataset was initially compared with individual bacterial strains at their respective minimum inhibitory concentrations, followed by comparisons with the positive control group at varying concentrations. Statistical significance was determined as p***< 0.001, p**< 0.01, and p*< 0.05 (n = 3).


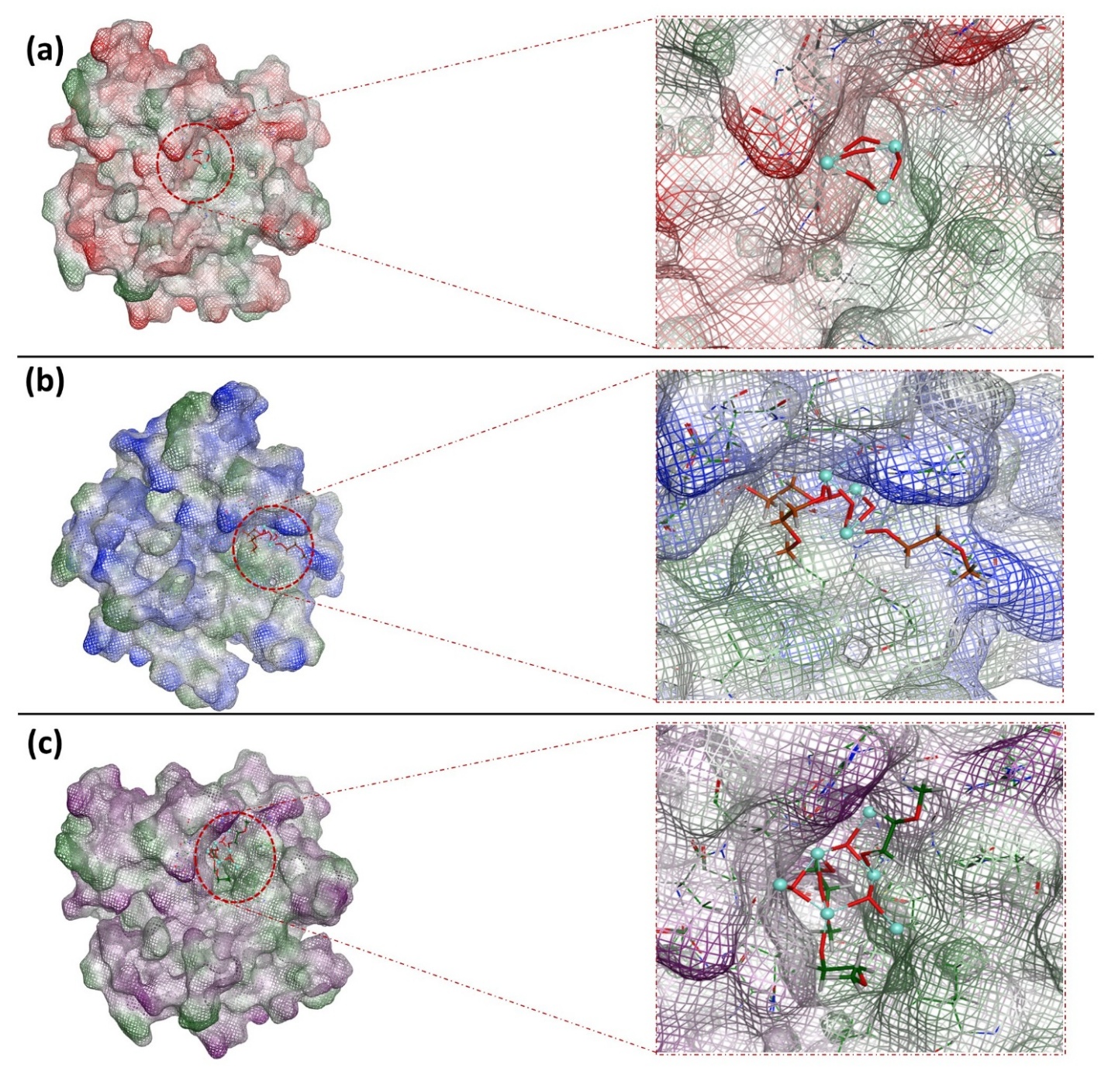


Figure S7 3D structural illustration of all three samples: (a) Fe_3_O_4_, (b) Fe_3_O_4_@mPEG, and (c) Fe_3_O_4_@mPEG-Ag inside active packets of *DNA-gyrase^(S. aureus^*^)^ complex.

Table S2 Comparative antibacterial efficacy of Fe_3_O_4_@mPEG-Ag hybrid-NPs and literatures reported nanomaterials.

| **Materials** | **MIC (µg**ꞏ**mL^-1^)** | **Effectiveness** | **Pathogens** |
| --- | --- | --- | --- |
| Fe_3_O_4_[1] | ----- | active | *S. aureus, E. coli* |
| Fe_3_O_4_@PVA[12] | 100 | active | *E. coli, P. aeruginosa* |
| Fe_3_O_4_@PEG@Gen[2] | 100 | active | *A. baumannii, P. mirabilia* |
| Fe_3_O_4_@GO[3] | 300 | active | *E. coli, S. aureus* |
| Ag_0.5_Fe_2.5_O_4_[4] | ----- | active | *C. albican* |
| Fe_3_O_4_@Ag[5] | 100 | active | *S. aureus, E. coli,* |
| G-Fe_3_O_4_@TiO_2_[47] | 100-200 | active | *E. coli, S. aureus* |
| Fe_3_O_4_@SiO_2_@PDA@Ag[6] | 115 | active | *subtitle, P. aeruginosa* |
| Fe_3_O_4_@CMC[7] | 58-300 | active | *S. aureus, P. aeruginosa*  *S. aureus, E. coli* |
| Fe_3_O_4_@C@Ag[20] | ----- | active | *E. coli* |
| MXene@Fe_3_O_4_/PDA[8] | 120 | active | *E. coli, S. aureus* |
| Fe_3_O_4_@PDA@Ag@PDA[9] | 200 | active | *E. coli, S. aureus* |
| Fe_3_O_4_@mPEG-Ag  (This work) | 50 | active | *S. aureus, E. faecalis, E. coli and K. pneumonaie* |

**References**

[1] E.N. Hammad, S.S. Salem, A.A. Mohamed, W. El-Dougdoug, Environmental Impacts of Ecofriendly Iron Oxide Nanoparticles on Dyes Removal and Antibacterial Activity, Appl Biochem Biotechnol 194 (2022) 6053–6067. https://doi.org/10.1007/s12010-022-04105-1.

[2] F.M. Abdulsada, N.N. Hussein, G.M. Sulaiman, A. Al Ali, M. Alhujaily, Evaluation of the Antibacterial Properties of Iron Oxide, Polyethylene Glycol, and Gentamicin Conjugated Nanoparticles against Some Multidrug-Resistant Bacteria, J Funct Biomater 13 (2022) 138. https://doi.org/10.3390/jfb13030138.

[3] A. N. Mahmoodabadi, A. Kompany, M. Mashreghi, Characterization, antibacterial and cytotoxicity studies of graphene-Fe_3_O_4_ nanocomposites and Fe_3_O_4_ nanoparticles synthesized by a facile solvothermal method, Mater Chem Phys 213 (2018) 285–294. https://doi.org/10.1016/j.matchemphys.2018.04.033.

[4] A.A.H. El-Bassuony, H.K. Abdelsalam, Attractive study of the physical properties of silver iron oxide nanoparticles for biomedical applications, Phys Scr 98 (2023) 055919. https://doi.org/10.1088/1402-4896/acc90c.

[5] F.A.M. Al-Zahrani, N.A. AL-Zahrani, S.N. Al-Ghamdi, L. Lin, S.S. Salem, R.M. El-Shishtawy, Synthesis of Ag/Fe_3_O_4_ nanocomposite from essential oil of ginger via green method and its bactericidal activity, Biomass Convers Biorefin 14 (2024) 13265–13273. https://doi.org/10.1007/s13399-022-03248-9.

[6] S. Singh, T. Goel, A. Singh, H. Chugh, N. Chakraborty, I. Roy, M. Tiwari, R. Chandra, Synthesis and characterization of Fe_3_O_4_@SiO_2_@PDA@Ag core–shell nanoparticles and biological application on human lung cancer cell line and antibacterial strains, Artif Cells Nanomed Biotechnol 52 (2024) 46–58. https://doi.org/10.1080/21691401.2023.2295534.

[7] M.H. Pourrafsanjani, R. Taghavi, A. Hasanzadeh, S. Rostamnia, Green stabilization of silver nanoparticles over the surface of biocompatible Fe_3_O_4_@CMC for bactericidal applications, Int J Biol Macromol 277 (2024) 134227. https://doi.org/10.1016/j.ijbiomac.2024.134227.

[8] J. Jin, S. Wu, X. Fang, H. Li, J. Wang, S. Xuan, W. Kong, D. Wang, X. Chen, K.C.-F. Leung, Q. Fang, T. Luo, MXene@Fe_3_O_4_/PDA nanosheets with photothermal-magnetically coupled antibacterial properties, Mater Chem Phys 322 (2024) 129562. <https://doi.org/10.1016/j.matchemphys.2024.129562>.

[9] Q. Fang, K. Xu, J. Zhang, Q. Xiong, J. Duan, S. Xuan, Hybrid Polydopamine/Ag Shell-Encapsulated Magnetic Fe_3_O_4_ Nanosphere with High Antibacterial Activity, Materials 13 (2020) 3872. https://doi.org/10.3390/ma13173872.
